# Adapting cytoskeleton-mitochondria patterning with myocyte differentiation by promyogenic PRR33

**DOI:** 10.1038/s41418-024-01363-w

**Published:** 2024-08-15

**Authors:** Xuyang Fu, Feng Zhang, Xiaoxuan Dong, Linbin Pu, Yan Feng, Yang Xu, Feng Gao, Tian Liang, Jianmeng Kang, Hongke Sun, Tingting Hong, Yunxia Liu, Hongmei Zhou, Jun Jiang, Deling Yin, Xinyang Hu, Da-Zhi Wang, Jian Ding, Jinghai Chen

**Affiliations:** 1 Department of Cardiology of Second Affiliated Hospital, State Key Laboratory of Transvascular Implantation Devices, Heart Regeneration and Repair Key Laboratory of Zhejiang Province, Zhejiang University School of Medicine, Hangzhou, 310009 China; 2 Institute of Translational Medicine, Zhejiang University School of Medicine, Hangzhou, 310029 China; 3 School of Life Science and Technology, Xi’an Jiaotong University, Xi’an, 710049 China; 4 Department of Cardiology, Ren Ji Hospital, School of Medicine, Shanghai Jiao Tong University, Shanghai, 200127 China; 5 University of South Florida Health Heart Institute, Center for Regenerative Medicine, Morsani College of Medicine, University of South Florida, Tampa, FL 33602 USA

**Keywords:** Development, Metabolic pathways

## Abstract

Coordinated cytoskeleton-mitochondria organization during myogenesis is crucial for muscle development and function. Our understanding of the underlying regulatory mechanisms remains inadequate. Here, we identified a novel muscle-enriched protein, PRR33, which is upregulated during myogenesis and acts as a promyogenic factor. Depletion of *Prr33* in C2C12 represses myoblast differentiation. Genetic deletion of *Prr33* in mice reduces myofiber size and decreases muscle strength. The *Prr33* mutant mice also exhibit impaired myogenesis and defects in muscle regeneration in response to injury. Interactome and transcriptome analyses reveal that PRR33 regulates cytoskeleton and mitochondrial function. Remarkably, PRR33 interacts with DESMIN, a key regulator of cytoskeleton-mitochondria organization in muscle cells. Abrogation of PRR33 in myocytes substantially abolishes the interaction of DESMIN filaments with mitochondria, leading to abnormal intracellular accumulation of DESMIN and mitochondrial disorganization/dysfunction in myofibers. Together, our findings demonstrate that PRR33 and DESMIN constitute an important regulatory module coordinating mitochondrial organization with muscle differentiation.

## Introduction

Skeletal muscle, accounting for about 40% of animal body mass, is the most prevalent tissue in vertebrates and plays a vital role in maintaining posture and controlling locomotion [1]. The development and homeostasis of skeletal muscle highly rely on the complex myogenic process involving myoblast differentiation and myofiber formation [2–5]. Dysregulated myogenesis can compromise muscle function and represent one of the major causes of skeletal muscle disorders. Therefore, investigating the mechanisms governing myogenesis holds significant promise for developing therapeutic interventions to treat myopathies [4, 6–8]. Myogenesis entails numerous inter- and intra-cellular regulatory events modulated by a wide array of factors. The downstream intracellular pathways induced by myogenic signals ultimately converge on the specific transcriptional/epigenetic regulators (MYOD, MYF5, MYOGENIN and MEF2 etc.) [4, 9, 10], which hierarchically orchestrate gene expression programs within cells. Furthermore, additional regulatory events, including the coordinated alterations of cell morphology and extracellular matrix structure, as well as cytoskeleton and subcellular organelle dynamics, are integral to myoblast differentiation and myofiber formation [11–13]. The elaborate interplay of these processes contributes to the establishment and maintenance of  terminal myogenic phenotypes [1, 14, 15].

Mitochondria are multifaceted organelles that exert diverse regulatory roles in myogenesis [16–18]. During this process, these organelles undergo a series of alterations in their content, morphology, and intracellular distribution, facilitating the adaption to changes in energy demands and aiding in myoblast commitment and differentiation [16, 18]. Notably, mitochondria also intimately modulate myogenic signaling and muscle gene expression in the cells, by upregulating pro-myogenic factors (MYOGENIN and MEF2 etc.) and/or repressing anti-myogenic ones (c-MYC etc.) [19–21]. Proper intracellular organization of mitochondria is crucial for myogenesis and muscle homeostasis, and the disruption of their organization is often a hallmark of muscle disorders [21, 22]. Establishing, coordinating and maintaining their spatial patterns in muscle cells, thus, is evidently of physiological importance.

Within skeletal myofibers, mitochondria are mainly associated with and presented at the Z-disc of the sarcomere along the length of each myofibril [23]. The juxtaposition and coupling of mitochondria with the sarcomeres appear to be very crucial for the optimized energy production and consumption [24]. The muscle-specific intermediate filament protein DESMIN is localized around the Z-disc and has been proposed to act as a link between mitochondria and myofibrils [25–27]. Loss of DESMIN activity in myocytes results in decreased number and mis-localization of mitochondria, causing mitochondrial dysfunction and muscle differentiation defects [28–30]. Mutations of *DES* gene in human impairs both cardiac and skeletal muscle function, leading to myopathies [31, 32]. Recently, research on DESMIN-regulated mitochondrial organization in skeletal muscle has been expanding. Current insights were mainly derived from investigations on DESMIN regulators and effectors. Various factors, such as MTM1 and DRP1, have been discovered to interact with DESMIN and regulate intermediate filament (IF) architecture as well as intracellular mitochondrial distribution in skeletal muscle cells [33, 34]. Nevertheless, our comprehension of this essential process remains inadequate. Particularly, factors within the regulatory networks are largely unexplored, and pathways responsible for coordinating mitochondrial organization with myogenesis remain poorly defined.

In this study, we identified PRR33, a skeletal muscle-enriched protein, as a pro-myogenic factor required for normal muscle differentiation and performance. Our findings demonstrate that PRR33 interacts with and may act upstream of DESMIN in regulating mitochondrial organization and function. Our study identifies a new protein coordinating DESMIN-mediated mitochondrial organization in skeletal muscle, which may hold the potential as a promising therapeutic target for muscle disorders.

## Results

**Fig. 1:**
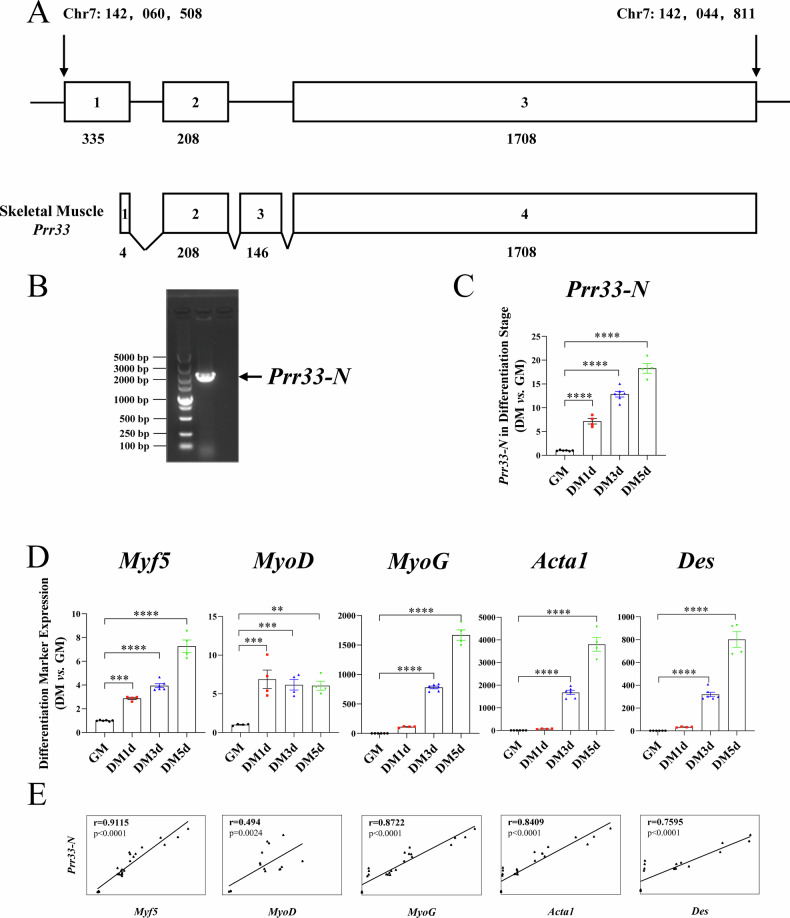
*Prr33* is upregulated during myoblasts differentiation. **A** Schematic for mouse *Prr33* genomic locus and skeletal muscle mRNA isoforms. **B** Full-length (2066 bp) transcript of *Prr33* (*Prr33-New* or *Prr33-N*) obtained from RNA isolated from the tibialis anterior muscle of adult mouse. **C** The relative levels of *Prr33-N* mRNA in C2C12 during differentiation. GM growth medium, DM differentiation medium. The data was presented as means ± SEM in the graph (One-way ANOVA). GM: *N* = 6, DM1d: *N* = 4, DM3d: *N* = 6, DM5d: *N* = 4. **D** Expression of myogenesis markers during C2C12 differentiation. The data was presented as means ± SEM (One-way ANOVA). GM: *N* = 6, DM1d: *N* = 4, DM3d: *N* = 6, DM5d: *N* = 4. **E** Expression of *Prr33-N* was positively correlated with those of myogenic genes. Pearson’s correlation test (r,95% confidence interval [CI]).

The notion of tissue-preferential expression is central to the identification of key genes regulating specific physiological processes of interest. We have been characterizing new factors modulating muscle development and function [35–37], and here found that a previously undefined gene *PRR33* (*Proline rich 33*) is highly expressed in human skeletal muscle (Human Protein ATLAS HPA dataset) (Fig. S1A). In mouse, the gene encoding PRR33 is located on chromosome 7 (Fig. 1A). We performed 3’ and 5’ rapid amplification of cDNA end (RACE) assays and cloned the full-length transcript of *Prr33* (Fig. 1B) using RNA isolated from adult mouse tibialis anterior (TA) muscle. The resulting full-length sequence (*Prr33-New*, or *Prr33-N*) spans approximately 2.1 kb, which, notably, stands as a unique transcript distinct from the annotated isoforms (*Prr33-201*, *Prr33-202*, *Prr33-203*) cataloged in the UniProt database (Fig. S1B). It appears to be the predominant isoform in skeletal muscle tissue, as indicated by its exclusive detection in our reverse transcription PCR assay (Fig. 1B). The coding region of the sequence exhibits high conservation across mammalian species (*PRR33-Human* and *Prr33-N* share 67.4% sequence identity; *Prr33-Rat* and *Prr33-N* share 87.14% sequence identity; *PRR33-Human* and *Prr33-Rat* share 68.09% sequence identity).

We assessed the mRNA expression level of *Prr33* across various tissues with isoform-specific primers, revealing that in general they are highly enriched in skeletal muscle and heart tissues, except that *Prr33-202* displays relatively consistent mRNA levels across diverse tissues (Fig. S1C–F). The skeletal muscle-preferential expression pattern of *Prr33* in mouse was similar to what was observed in human samples (Human Protein ATLAS, HPA dataset) (Fig. S1A). This study primarily focuses on the function of mouse *Prr33* in skeletal muscle. We conducted qRT-PCR assays on mouse muscle tissue samples employing isoform-specific primers, and found that the newly identified 2.1 kb isoform (*Prr33-N*) was notably more abundant than others (Fig. S1G–K).

*Prr33* is partially localized in the antisense DNA strand within the genomic locus of the *Tnnt3*, which encodes a fast-twitch troponin protein. Strong positive correlations in expression between genes and their antisense transcripts have been frequently observed in previous studies [38, 39]. Given this, we asked whether the gene *Prr33* could be preferentially expressed in coordination with *Tnnt3* in fast-twitch muscle fibers. To test this idea, we conducted an analysis of mRNA levels of *Prr33-N* across different muscle types (Triceps muscle: TRI, Quadriceps femoris muscle: QUAD, Gastrocnemius muscle: GAS, Tibialis anterior muscle: TA and Soleus muscle: Soleus). Indeed, *Prr33-N* displays a similar expression pattern to *Tnnt3* in muscle tissues. (Fig. S1L, M).

Next, we assessed the level of the *Prr33-N* transcript in C2C12 cells. Interestingly, during C2C12 myoblast differentiation, the mRNA expression of *Prr33-N* is elevated (Fig. 1C) and exhibits co-upregulation with the markers of muscle myogenesis, including *Myogenic factor 5* (*Myf5)*, *Myogenic differentiation1* (*MyoD*), *Myogenin* (*MyoG*), *Skeletal muscle actin α 1 (Acta1)* and *Desmin* (*Des*) (Fig. 1D, E).

### PRR33 positively regulates myoblast differentiation

To investigate the role of PRR33 in skeletal muscle function, we knocked down *Prr33* gene (*Prr33*-KD) in C2C12 cells using Lenti-shRNA (*shprr33*) and evaluated the impact of *Prr33*-KD on both myoblast proliferation and differentiation. For the C2C12 cells cultured in regular growth medium supplied with 20% fetal bovine serum (FBS), knockdown of *Prr33* appeared not to affect cell density (Fig. S2A). The cells were also stained with the cell-cycle and proliferative marker EdU (5-ethynyl-2′-deoxyuridine). We quantified the percentage of EdU^+^ cells and found no significant difference between *Prr33*-KD myoblasts and control cells (Fig. S2A, B). Expression of cell cycle genes was assayed with qRT-PCR, yet no significant alteration was observed when *Prr33* was knocked down (Fig. S2C). Together, depletion of *Prr33* appeared not to affect myoblast proliferation. Next, we induced differentiation by switching the culture to low-serum medium (2% horse serum). As a result, the majority of control cells underwent myogenesis and formed multi-nucleated myotubes expressing MYH1E (Myosin heavy chain 1 E, differentiation marker, stained by MF20 antibody) within approximately three days (D3). However, a significantly lower number of multiple nuclei myotubes was observed in *Prr33*-KD C2C12 cells (Fig. S2D, E), and the length of MF20 positive myotube was shorter in *Prr33*-KD C2C12 group (Fig. S2F), indicating that *Prr33* inhibition suppressed myoblast differentiation. We also analyzed the expression levels of *Myogenic factor 5* (*Myf5)*, *Myogenic differentiation1* (*MyoD*), *Myogenin* (*MyoG*), *Skeletal muscle actin α 1 (Acta1)* and *Desmin* (*Des*) which are myogenic differentiation marker genes, and found that most of them were substantially downregulated by *Prr33* knockdown (Fig. S2G).

**Fig. 2:**
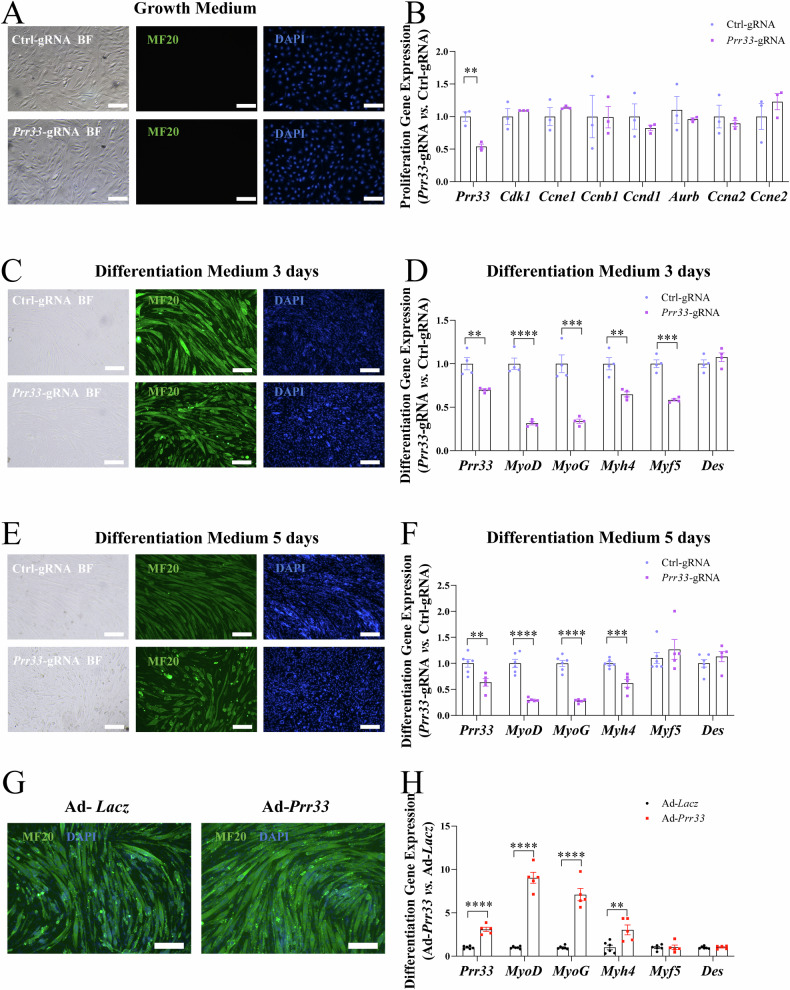
PRR33 positively regulates myoblast differentiation. **A** Representative images of C2C12 myoblasts cultured in growth medium expressing Cas9/CRISPR-gRNA targeting *Prr33* gene (*Prr33*-gRNA) or Cas9/CRISPR-ctrl-gRNA (Ctrl-gRNA) with immunofluorescence staining for MYH1E (MF20, green) and DAPI (blue). BF: bright filed. Bar: 100 μm. **B** Expression of *Prr33* and proliferation markers (*Cdk1*, *Ccne1* and *etc*.) in C2C12 myoblasts was assessed by qRT-PCR. Data was presented as mean ± SEM. (Student’s *t-test*). *N* = 3. **C** Representative images of C2C12 cells at differentiation day 3 (D3) with immunofluorescence staining for MYH1E (MF20, green) and DAPI (blue). BF: bright filed. Bar: 100 μm. **D** The expression of *Prr33* and differentiation markers at differentiation day 3 was assessed by qRT-PCR. Data was presented as mean ± SEM. (Student’s *t-test*). *N* = 4. **E** Representative images of C2C12 cells (differentiation day 5, D5) with immunofluorescence staining for MYH1E (MF20, green) and DAPI (blue). BF: bright filed. Bar: 100 μm. **F** The expression of *Prr33* and differentiation markers at differentiation day 5 was assessed by qRT-PCR. Data was presented as mean ± SEM. (Student’s *t-test*). *N* = 6. **G** Representative images of C2C12 myoblasts expressing exogenous *Prr33* (Ad-*Prr33*) or Lacz (Ad-*Lacz* as control) at differentiation day 3 (D3) with immunofluorescence staining for MYH1E (MF20, green) and DAPI (blue). Bar: 100 μm. **H** The expression of *Prr33* and differentiation markers at differentiation day 3 was assessed by qTR-PCR. Data was presented as mean ± SEM. (Student’s *t-test*). Ad-*Lacz*: *N* = 6, Ad-*Prr33*: *N* = 5.

We next created another loss-of-function model in C2C12 cells by introducing Cas9/CRISPR-gRNA to target the *Prr33* gene (*Prr33*-gRNA-treated). As shown in EdU and Ki67 (antigen identified by monoclonal antibody Ki67, proliferative marker) staining assays, the genetic ablation of *Prr33* had no influence on C2C12 proliferation when cultured in growth medium (Figs. 2A, B; S3A–D), while a reduction in both the number and the length of the formed myotubes (Fig. S3E–H), as well as a decrease in the expression of myogenic markers was consistently observed in *Prr33* loss-of-function cells (Figs. 2C–F; S3I, J).

*Prr33* is partially localized in the antisense DNA strand within the genomic locus of the *Tnnt3*, encoding the fast-skeletal muscle TnT. Antisense transcripts have been discovered to regulate the expression of their corresponding sense genes [40]. Therefore, we sought to investigate whether the depletion of *Prr33* could influence the expression of *Tnnt3* and other myofiber type-specific markers. We examined the expression of fast-twitch (*Tnnt3*, *Tpm2*, *Myl1*, *Myl9*) and slow-twitch (*Tnnc1*, *Myl3*, *Myh7b*), in two *Prr33* loss-of-function models (*Prr33*-KD and *Prr33*-gRNA). However, no significant alterations of these markers were detected (Figs. S2H; S3K).

Notably, inhibition of *Prr33* apparently also led to reduced size of the myotubes. We sought to investigate whether this reduction stemmed from suppressed differentiation or muscle cell atrophy, characterized by cellular shrinkage [41]. To explore the possibility, we treated C2C12 cells with *Prr33* siRNA on differentiation day 2(D2), a stage where majority of the myoblasts had already differentiated into myocytes and formation of myotubes had been occurring. Control cells transfected with scramble siRNA (si-NC) continued to differentiate, with over>99% of cells were labeled by the myogenic marker (MF20 antibody). In contrast, approximately 15% of *Prr33* siRNA-treated C2C12 cells were MF20-negative (*p* < 0.0001, Fig. S4A, B), indicating that *Prr33* siRNA treatment at D2 impeded the differentiation process. Intriguingly, the size of the formed myotubes (MF20-positive) in *Prr33* siRNA-treated group appeared to be indistinguishable from that of the control cells (Fig. S4C), and the expression of atrophy marker genes (*Fbxo32*,*Trim63*, and *Myostatin*) was not altered by *Prr33* siRNA treatment at D2 (Fig. S4D), while the expression of differentiation marker genes (*MyoD* and *MyoG*) was inhibited (Fig. S4D), indicating that the observed decrease in myotube size (Figs. 2C–F; S2D–F) was mainly due to suppressed differentiation.

Next, we investigated whether the upregulation of *Prr33* could influence myoblast differentiation. We prepared an adenovirus to overexpress *Prr33* in C2C12 myoblasts and examined the gain-of-function effects (Fig. S5A, B). Exogenous expression of *Prr33* appeared to enhance the myogenic differentiation of the myoblasts, with more myotubes formed in Ad-*Prr33* cells. The myotubes derived from Ad-*Prr33*-expressing C2C12 were larger and contained a greater number of nuclei than those from control cells (Figs. 2G; S5C, D). The expression levels of myogenic markers such as *MyoG*, *MyoD*, and *Myh*4 were also upregulated following Ad-*Prr33* treatments (Figs. S5A, B; 2H). Taken together, our results from both loss-of-function and gain-of-function assays consistently demonstrate that PRR33 is a critical pro-myogenic regulator of myoblast differentiation.

### Loss of PRR33 reduces the size of myofibers in vivo and results in a decrease in muscle strength

**Fig. 3:**
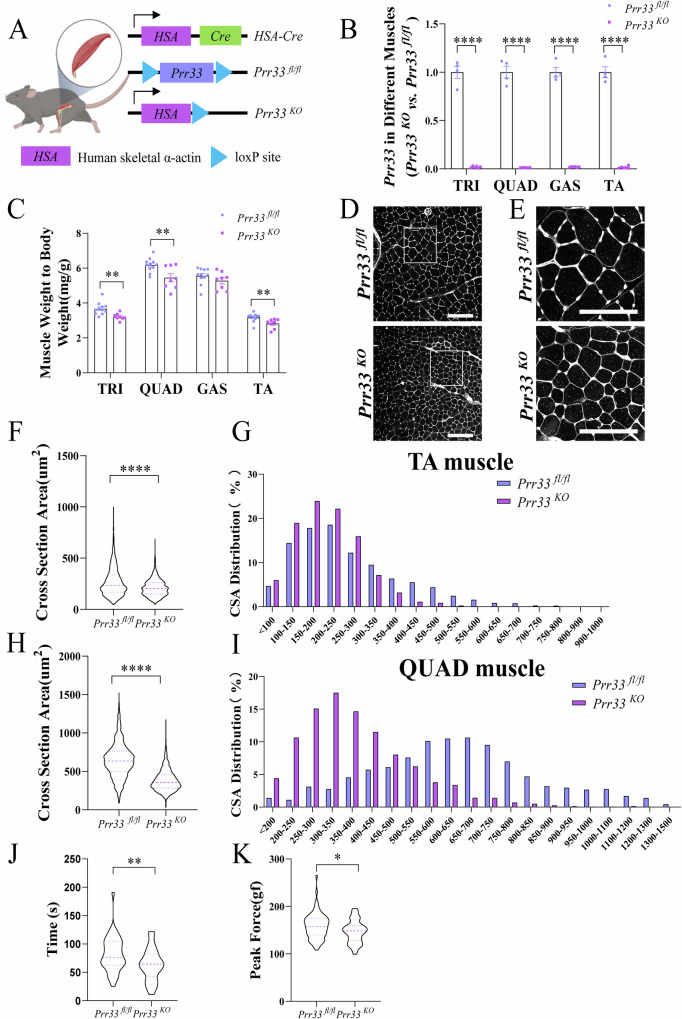
Loss of PRR33 reduces the size of myofibers in vivo and results in a decrease in muscle strength. **A** The genetic model in which *Prr33* was abrogated in skeletal muscle. *HSA*: Human skeletal α-actin. **B** mRNA level of *Prr33* in mouse muscle samples including TRI, QUAD, GAS and TA assessed by qRT-PCR. TRI Triceps muscle, QUAD: Quadriceps femoris muscle, GAS Gastrocnemius muscle, TA Tibialis anterior muscle. Data was presented as mean ± SEM. (Student’s *t-test*). *Prr33*^*fl/fl*^: *N* = 4; *Prr33*^*KO*^: *N* = 6. **C** The ratio of muscle weight to body weight in *Prr33*^*fl/fl*^ and *Prr33*^*KO*^ mice. Data was presented as mean ± SEM. (Student’s *t-test*). *Prr33*^*fl/fl*^: *N* = 11; *Prr33*^*KO*^: *N* = 8. **D** WGA staining of TA muscle section of *Prr33*^*fl/fl*^ and *Prr33*^*KO*^ mice (4-week-old). *Prr33*^*fl/fl*^: *N* = 7; *Prr33*^*KO*^: *N* = 5. Bar: 150 μm. **E** Zoomed-in images of the regions (white squares) showed in Fig. 3D. Bar: 20 μm. **F** Myofiber size in the cross-section areas (CSA) of TA myofibers from *Prr33*^*fl/fl*^ or *Prr33*^*KO*^ mice were analyzed at 4 weeks after birth using ImageJ software (https://imagej.nih.gov/ij/). *Prr33*^*fl/fl*^: *N* = 7; *Prr33*^*KO*^: *N* = 5. **G** Myofiber size distribution in the cross-section areas of TA muscle in *Prr33*^*fl/fl*^ and *Prr33*^*KO*^ mice at 4 weeks after birth. *Prr33*^*fl/fl*^: *N* = 7; *Prr33*^*KO*^: *N* = 5. Myofiber size was measured with ImageJ. **H** Myofiber size in the cross-section areas (CSA) of QUAD myofibers from *Prr33*^*fl/fl*^ and *Prr33*^*KO*^ mice were analyzed at 4 weeks after birth using ImageJ software (https://imagej.nih.gov/ij/). *Prr33*^*fl/fl*^: N = 7; *Prr33*^*KO*^: *N* = 5. **I** Myofiber size distribution of the cross-section areas of QUAD muscle in *Prr33*^*fl/fl*^ and *Prr33*^*KO*^ mice at 4 weeks after birth. *Prr33*^*fl/fl*^: *N* = 7; *Prr33*^*KO*^: N = 5. Myofiber size was measured with ImageJ. **J** Muscle endurance analysis for *Prr33*^*fl/fl*^ and *Prr33*^*KO*^ mice by running wheel assay. Endurance time data was documented and presented as mean ± SEM in the graph. (Student’s *t-test*). *Prr33*^*fl/fl*^: *N* = 4; *Prr33*^*KO*^: *N* = 4. **K** Limb grip strength measurement of *Prr33*^*fl/fl*^ and *Prr33*^*KO*^ mice. Data was presented as mean ± SEM. (Student’s *t-test*). *Prr33*^*fl/fl*^: *N* = 4; *Prr33*^*KO*^: *N* = 4.

To investigate the in vivo effects of *Prr33* ablation in skeletal muscle, we generated *HSA-Cre*; *Prr33*^*fl/fl*^ mice (*Prr33*^*KO*^), in which the *HSA-Cre*, expressing Cre recombinase driven by the skeletal muscle-specific *HSA* (Human Skeletal α-Actin) promoter, mediated the abrogation of the *Prr33* gene in skeletal muscle cells (Fig. 3A). We confirmed that the abolishment of *Prr33* expression in the skeletal muscle tissues, including Triceps muscle (TRI), Quadriceps femoris muscle (QUAD), Gastrocnemius muscle (GAS), and Tibialis anterior muscle (TA), of *Prr33*^*KO*^ mice (Fig. 3B). We observed a significantly reduction in the ratio of muscle weight (TRI, QUAD, and TA) to body weight in 4-week-old *Prr33*^KO^ mice (Fig. 3C). Wheat Germ Agglutinin (WGA) staining revealed that the myofibers in the mutants were generally thinner compared to those in control animals (Fig. 3D, E). Furthermore, the fiber size distribution was skewed towards smaller myofibers in the TA muscle of *Prr33*^*KO*^ mice (Fig. 3F, G), a trend consistently observed in the QUAD muscle tissues as well (Fig. 3H, I). Expression levels of myogenic markers in *Prr33*^*KO*^ muscle tissue samples were significantly downregulated (Fig. S6A–E), similar to what was observed in C2C12 cells. Subsequently, we evaluated the motor ability of the mutant mice and found that the athletic performance of *Prr33*^*KO*^ mice was significantly poorer than that of the control mice. *Prr33*^*KO*^ mice demonstrated reduced endurance during continuous motion on the wheel (Fig. 3J) and exhibited lower peak force of grip compared to control mice (Fig. 3K).

Skeletal muscle has the ability to regenerate in response to injury, and myoblast differentiation is a crucial part of the process [42, 43]. Deficiencies in myogenic differentiation can impede the repair of muscle tissue [44, 45]. To investigate the functional consequence of PRR33-regulated myogenesis on muscle regeneration, we induced muscle injury in *Prr33*^*KO*^ and control (*Prr33*^*fl/fl*^) mice by injecting barium chloride (BaCl_2_, a potassium channel blocker, which can induce myonecrosis in muscle) into their TA muscles [46]. Substantial up-regulation of *Prr33* in the injured tissue was observed 7 and 14 days after injection (Fig. S7A). We monitored muscle regeneration at the different time points post-injury. During muscle regeneration, newly formed myofibers express the embryonic isoform of myosin heavy chain (eMHC), which distinguishes them from pre-existed myofibers. The re-expression of eMHC serves as a specific marker of in vivo muscle differentiation and regeneration [46, 47]. In control mice, eMHC was significantly elevated on day 3 after BaCl_2_ injection (Fig. S7B, Saline *Prr33*^*fl/fl*^ *versus* BaCl_2_ *Prr33*^*fl/fl*^). However, the injury-induced upregulation of eMHC was inhibited in *Prr33*^*KO*^ mice (Fig. S7B, BaCl_2_ *Prr33*^*fl/fl*^ *versus* BaCl_2_ *Prr33*^*KO*^). As shown in Fig. S7C, compared to *Prr33*^*fl/fl*^ mice, *Prr33*^*KO*^ mice exhibited relatively lower expression of eMHC. Notably, at day 7 and 14 post injury, the cross-section-areas of myofibers in *Prr33*^*KO*^ mice were smaller than those in control mice (Fig. S7D, E), suggesting that *Prr33* ablation impairs muscle differentiation during regeneration. These findings were further supported by H&E staining analysis (Fig. S7F). Furthermore, the muscle size of *Prr33*^*KO*^ mice was significantly smaller than that of control mice after 14 days of recovery (Fig. S7G). Overall, our results consistently demonstrate that PRR33 is required to the proper muscle differentiation and regeneration, underscoring its role as a key regulator of skeletal muscle development and function.

### Genes encoding cytoskeleton and mitochondrial factors are deregulated by *Prr33* depletion in muscle cells

**Fig. 4:**
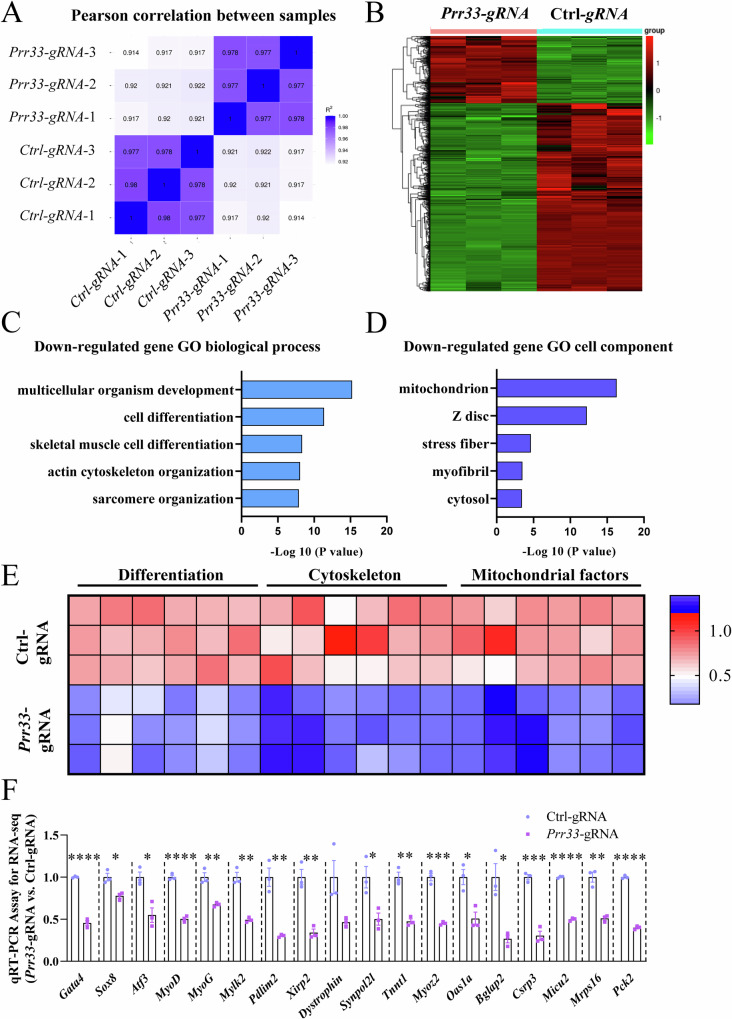
Genes encoding cytoskeleton and mitochondrial factors are deregulated by *Prr33* depletion in muscle cells. **A** Heatmap showing inter-sample correlations of gene profiles. **B** Hierarchical heatmap of differentially expressed genes at differentiation day3, (log2 FC > 1; *P* < 0.05). *N* = 3. **C** Gene Ontology-biological process (GO-BP) analysis of downregulated genes according to -Log10 *P* value. **D** Gene Ontology-cell component (GO-CC) analysis of downregulated genes according to -Log10 P value. **E** Heatmaps of differentially expressed genes related to differentiation, cytoskeleton and mitochondrial factors in *Prr33*-gRNA-treated C2C12 myotubes. **F** qRT-PCR assay for the genes related to differentiation (Fig. 4C), cytoskeleton (Fig. 4C) and mitochondrial factors (Fig. 4D). *N* = 3.

RNA sequencing (RNA-seq) was performed on a *Prr33*-gRNA-treated myotubes (D3) to investigate the molecular events and pathways influenced by PRR33. The analysis revealed 1,704 differentially expressed genes (log2 FC > 1; *P* < 0.05) in *Prr33*-gRNA-treated cells compared to Ctrl-gRNA C2C12 cells, comprising 450 upregulated and 1,254 downregulated genes (Fig. 4A, B). The abundance of numerous transcripts related to “cell differentiation”, “skeletal muscle cell differentiation” were significantly decreased in *Prr33*-gRNA-treated myotubes. Such molecular pattern was consistent with the myogenic phenotypes observed in vitro and in vivo. The downregulated genes were grouped to other categories of biological processes including “actin cytoskeleton organization”, and “sarcomere organization” (Fig. 4C). Intriguingly, utilizing the cell compartments annotated in gene ontology (GO) encyclopedia, we found that many of the downregulated genes encode mitochondrial factors (Fig. 4D). We validated the expression of these genes by qRT-PCR and found that all tested representative candidates were downregulated when *Prr33* was abrogated, which is consistent with the RNA-seq results (Fig. 4E, F). Downregulation of these genes related to skeletal muscle differentiation, cytoskeleton and mitochondrial activity were also observed in the skeletal tissue samples of *Prr33*^*KO*^ genetic mice (Fig. S8A).

The interplay between cytoskeleton and mitochondria actively participates in modulating myogenesis and muscle function [23, 48, 49]. To meet the biomechanical and energetic requirements, regulation of mitochondria structure and function needs to be coordinated with cytoskeleton and sarcomere morphogenesis in muscle cells [23, 24]. Our data collectively suggests that PRR33 may be involved in this crucial process, and deregulation of these mitochondrial genes may be relevant to the myogenic defects observed in *Prr33*-KD and KO models.

### PRR33 modulates mitochondrial oxidative phosphorylation and morphology

**Fig. 5:**
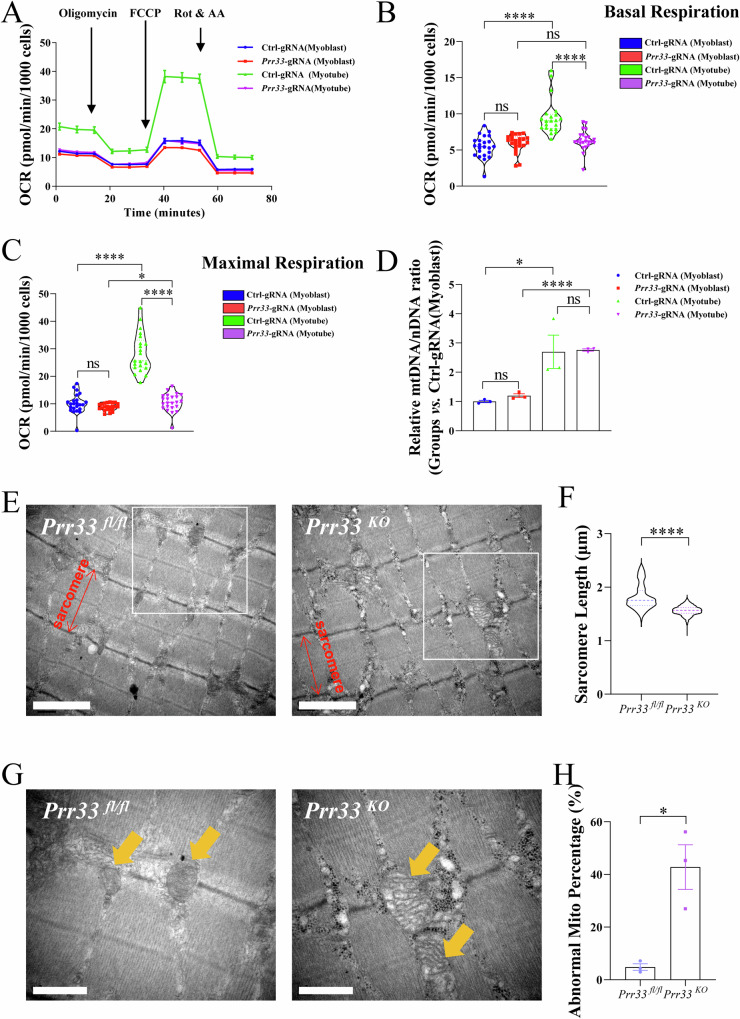
PRR33 modulates mitochondrial oxidative phosphorylation and morphology. **A** Mitochondrial respiration (Oxygen consumption rate, OCR) in different C2C12 cells. Blue group: Ctrl-gRNA (Myoblast), control-gRNA-treated C2C12 cultured in growth medium; Red group: *Prr33*-gRNA (Myoblast), *Prr33*-gRNA-treated C2C12 cultured in growth medium; Green group: Ctrl-gRNA (Myotube), control-gRNA-treated C2C12 cultured in differentiation medium for 3 days; Purple group: *Prr33*-gRNA (Myotube), *Prr33*-gRNA-treated C2C12 cultured in differentiation medium for 3 days. **B** Analysis of basal oxygen consumption rate (OCR). Ctrl-gRNA (Myoblast), *Prr33*-gRNA (Myoblast), Ctrl-gRNA (Myotube), *Prr33*-gRNA (Myotube). Data was presented as mean ± SEM. (Two-way ANOVA). **C** Analysis of maximal oxygen consumption rate (OCR). Ctrl-gRNA (Myoblast), *Prr33*-gRNA (Myoblast), Ctrl-gRNA (Myotube), *Prr33*-gRNA (Myotube). Data was presented as mean ± SEM. (Two-way ANOVA). **D** The relative abundance of mitochondrial DNA (mtDNA encoding *16SrRNA*) and nuclear DNA (nDNA encoding *Hexokinase 2*) in C2C12 cells was quantified with qRT-PCR. The mtDNA/nDNA ratio was calculated by following the ∆∆Ct method of qPCR assay. Data was presented as mean ± SEM. (Two-way ANOVA). **E** Representative Transmission Electron Microscopy (TEM) images of skeletal muscle samples. Red arrows: sarcomere. *N* = 3. Bar: 1.5 μm. **F** Sarcomere length was measured from TEM images with ImageJ and quantified as the distance between adjacent Z-lines. *N* = 3. Data was presented as mean ± SEM. (Student’s *t-test*). **G** Representative Transmission Electron Microscopy (TEM) images showing mitochondrial morphology. Zoomed-in images from the regions (white squares) in Fig. 5E were shown. Yellow arrows: mitochondrial. *N* = 3. Bar: 0.8 μm. **H** The percentiles of abnormal (swollen and cristae incompact) mitochondria to the total number of mitochondria observed in the view fields of TEM images. N = 3 (abnormal mitochondria number/total mitochondrial number: *Prr33*^*fl/fl*^ -1 (11/254); *Prr33*^*fl/fl*^ -2 (19/265); *Prr33*^*fl/fl*^ -3 (7/239); *Prr33*^*KO*^ -1 (78/172); *Prr33*^*KO*^ -2 (123/219); *Prr33*^*KO*^ -3 (65/241). Data was presented as mean ± SEM. (Student’s *t-test*).

Mitochondrial function boosted throughout normal myoblast differentiation, and oxidative phosphorylation is the major energy source during myogenesis [21]. We investigated whether depletion of *Prr33* affects mitochondrial function during myogenesis, by assessing the cellular respiration of control and *Prr33*-gRNA-treated cells during myoblast differentiation. The oxygen consumption rate (OCR) was analyzed using a Seahorse X96 Bioanalyzer, via the mitochondrial stress test, involving sequential addition of oligomycin, trifluoromethoxy carbonyl cyanide phenylhydrazone (FCCP), and antimycin + rotenone (A + R), to measure basal, maximal, and reserve respiration (Fig. 5A). Consistent with prior research, both basal and maximal oxygen consumption rates (OCRs) were significantly increased during differentiation in control cells [50]. However, in *Prr33*-gRNA-treated myoblasts, only a marginal elevation of the basal OCRs was observed in myogenesis. Mitochondrial oxidative phosphorylation was markedly reduced in *Prr33*-gRNA-treated myotubes compared to Ctrl-gRNA-treated myotubes (Fig. 5B, C). These findings demonstrate that inhibition of *Prr33* in myoblast impairs mitochondrial function during myoblast differentiation.

During myogenesis, a metabolic switch occurs to support the increased energetic demand [51]. The process involves mitochondrial repopulation and biogenesis [51–53]. We asked whether the impaired cellular respiration observed in *Prr33*-gRNA-treated cells could be attributed to alterations in mitochondrial biogenesis and quantity. Mitochondrial DNA to nuclear DNA ratio (mtDNA/nDNA), a marker of mitochondrial biogenesis [54], was measured, yet no significant difference was observed between *Prr33-*gRNA-treated and Ctrl-gRNA-treated cells (Fig. 5D). These analyses suggest that inhibition of *Prr33* may not affect mitochondrial quantity in muscle cells.

We employed Transmission Electron Microscopy (TEM) to examine sarcomere structure and the mitochondrial morphology in *Prr33*^*KO*^ skeletal muscle cells. The length of Z-line was not influenced by *Prr33* abrogation (Fig. S9A). Intriguingly, compared to control mice, the sarcomere length of mutants was significantly shorter (Fig. 5E, F). The observed shorter sarcomere length and thinner cross-section in *Prr33*^*KO*^ mice (Fig. 3D) further suggest that normal myofiber development was impaired by *Prr33* deletion. Additionally, we observed more abnormal (swollen and cristae incompact) mitochondria in the muscles of *Prr33*^*KO*^ mice (Fig. 5G, H). Moreover, the number and total length of cristae were decreased (Fig. S9B, C), while the area of mitochondria was increased (Fig. S9D) in *Prr33*^*KO*^ muscle. Together, our results demonstrate that PRR33 plays an important role in regulating mitochondrial morphology and function in muscle cells.

### PRR33 interacts with DESMIN

**Fig. 6:**
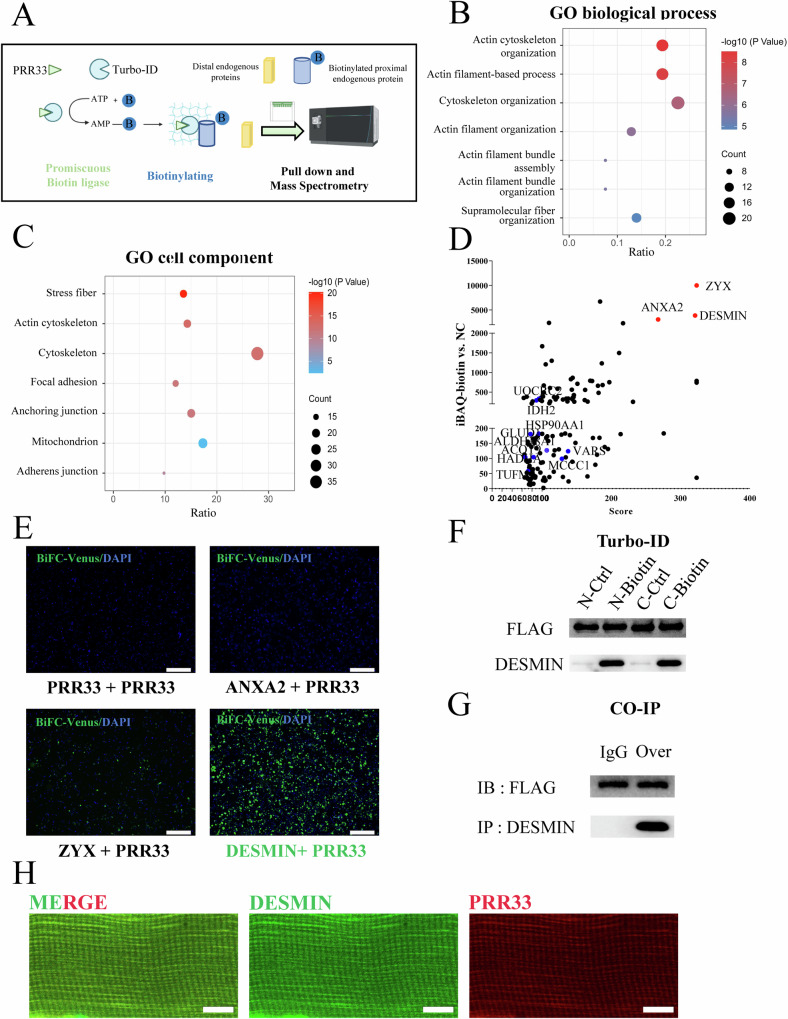
PRR33 interacts with DESMIN. **A** Schematic map of Turbo-ID-based proximal labeling and proteomics profiling. **B** Gene Ontology-biological process (GO-BP) enrichment analysis. *P* value < 0.05. **C** Gene Ontology-cell component (GO-CC) enrichment analysis. *P* value < 0.05. **D** Plot of proteins labeled by PRR33-Turbo-ID (iBAQ-biotin vs NC > 3000, Score>250). **E** BiFC-Venus fluorescence image of ANXA2, ZYX, DESMIN with PRR33. Bar: 400 μm. **F** Turbo-PRR33 (N) or PRR33-turbo (C) was introduced into C2C12 cells. The biotin-labeled proximal proteins were recovered by Streptavidin-beads and were subjected to Western Blot analysis with indicated antibodies. **G** Co-immunoprecipitation (Co-IP) experiments to verify the interaction between DESMIN and PRR33. IB FLAG represented the expression of PRR33 in both groups; IP DESMIN showed the interaction of IgG or PRR33 (Over). **H** Representative immunostaining image of wild type mouse TA muscle section. DESMIN (green), PRR33 (red). *N* = 3. Bar: 20 μm.

To unravel the potential molecular mechanism by which PRR33 regulates muscle differentiation and mitochondrial function, we profiled its neighboring proteins in C2C12 cells using Turbo-ID-based proximal labeling followed by Mass Spectra (Turbo-ID-MS) proteomic analysis (Fig. 6A) [55]. We identified 1313 proteins that were labeled by PRR33-Turbo-ID. Gene Ontology (GO) term enrichment analysis showed that these proteins were primarily related to “cytoskeleton organization”, “actin cytoskeleton organization”, “actin filament-based process”, “mitochondria” and “stress fiber” (Fig. 6B, C). Particularly, three cytoskeleton proteins from the category, ZYX (Zyxin), ANXA2 (Annexin A2) and DESMIN, were significantly enriched in PRR33-Turbo-ID samples. We thus narrowed our focus to three candidates (Fig. 6D).

We further investigated the putative interactions between PRR33 and the three candidates using bimolecular fluorescence complementation (BiFC) assay in living cells [56]. ZYX and DESMIN appeared to form complexes with PRR33. Particularly, we observed very strong fluorescence complementation between DESMIN and PRR33 (Fig. 6E). To corroborate these findings, we independently repeated the Turbo-ID experiment and confirmed the labeling of DESMIN by PRR33-Turbo-ID (Fig. 6F). Additionally, we conducted the Co-immunoprecipitation (Co-IP) experiments to verify the interaction between DESMIN and PRR33. Our Co-IP results unequivocally demonstrate that PRR33 is capable of forming complex(es) with DESMIN (Fig. 6G). Furthermore, we utilized immunostaining technology to confirm the localization of PRR33 and its co-localization with DESMIN in muscle cells (Fig. 6H).

The DESMIN protein is located within muscle sarcomere [57, 58]. By connecting Z-discs and mediating the interlink between neighbor sarcomere units, DESMIN facilitates the formation of myofibrils in muscle cells [30, 59, 60]. Notably, DESMIN is also a key regulator of muscle differentiation. Inhibition of DESMIN downregulates the myogenic HLH transcription factors (MYOD, MYF5 and MYOGENIN), and hinders the normal myogenic process [60]. We knocked down *Des* gene in C2C12 (si-*Des*), and observed repression in myoblast differentiation (Fig. S10A–F). Depletion of DESMIN also impaired mitochondrial function in the cells (Fig. S10G–I), similar to what we observed in PRR33 loss-of-function muscle cells. Taken together, our results suggest that DESMIN may participate in PRR33-regulated myoblast differentiation.

### *Prr33* ablation alters intracellular pattern of DESMIN and mitochondrial morphology

**Fig. 7:**
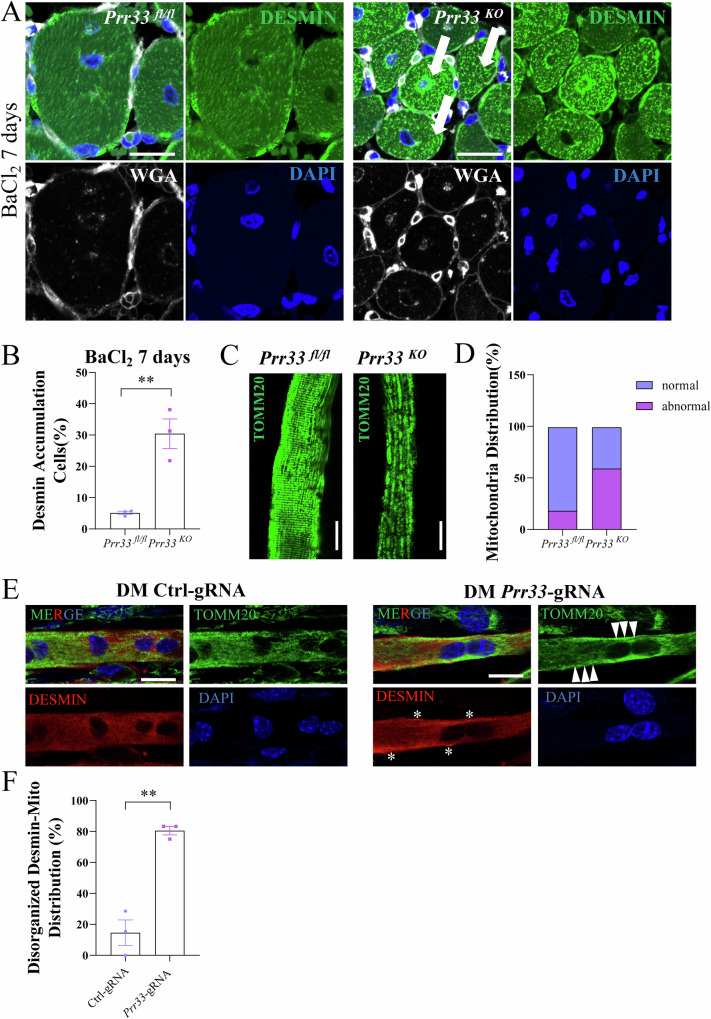
PRR33 ablation alters intracellular pattern of DESMIN and mitochondrial morphology. **A** Immunostaining images of *Prr33*^*fl/fl*^ or *Prr33*^*KO*^ TA muscle section at 7 days after BaCl_2_ injection. DESMIN (green), DAPI (blue), WGA (white). White arrows: accumulated DESMIN. *N* = 3. Bar: 40 μm. **B** Percentiles of myofibers showing DESMIN accumulation in the field in TA muscles at 7 days after BaCl_2_ injection. *N* = 3. Data was presented as mean ± SEM. (Student’s *t-test*). **C** Representative images of immunofluorescence staining for TOMM20 in *Prr33*^*fl/fl*^ and *Prr33*^*KO*^ TA myofibers. *N* = 3. Bar: 40 μm. **D** Distribution of myofibers with normal and abnormal intracellular pattern of mitochondria in *Prr33*^*fl/fl*^ and *Prr33*^*KO*^ TA muscle samples. *N* = 3. **E** Representative images of immunofluorescence staining for TOMM20 (green), DESMIN (red) and DAPI (blue) in C2C12 cells at differentiation day 3. Asterisk (*): accumulated DESMIN; White arrows: disorganized mitochondria. Bar: 30 μm. **F** Percentiles of myotubes with abnormal accumulated DESMIN and disorganized mitochondria in Ctrl-gRNA and *Prr33*-gRNA-treated samples.

The intracellular space contains 3 cytoskeletal systems: intermediate filament (IFs), microfilaments (MFs), and microtubules (MTs) [61, 62]. Mitochondria have been observed to associate with intermediate filaments in muscle cells [63, 64]. DESMIN represents the major cytoplasmic IF in skeletal muscle and can interact with mitochondria. Absence of DESMIN can alter mitochondria distribution and impair the respiration [28, 29, 49, 57, 64]. Given that PRR33 is a binding partner of DESMIN, and PRR33 can modulate mitochondrial function in muscle cells, we hypothesized that these molecular events may constitute an important regulatory module of myogenesis. To further assess their interplays, we monitored the intracellular patterns of DESMIN and mitochondria in *Prr33*^*KO*^ muscle cells. DESMIN is typically localized underneath the sarcolemma and at the level of Z-discs [34], connecting myofibrils to the sarcolemma at the level of the costamere. However, at 7 and 14 days after injury in *Prr33*^*KO*^ muscle cells, aggregation of DESMIN was observed (Figs. 7A, B; S11A, B, white arrows). Additionally, we examined the intracellular distribution of mitochondria in isolated myofibers from TA muscles of *Prr33*^*fl/fl*^ and *Prr33*^*KO*^ mice. *Prr33*^*fl/fl*^ myofibers exhibited well-organized mitochondria along the cytoskeleton, whereas *Prr33*^*KO*^ myofibers showed disordered distribution of mitochondria (Fig. 7C, D).

DESMIN directly interacts with mitochondria and has been proposed to act as a linkage to mediate the juxtaposition and coupling of mitochondria with the sarcomeres in skeletal muscle cells [49]. As shown in Fig. 6C, D, numerous mitochondrial proteins, including TUFM [65, 66], were labeled by PRR33-Turbo-ID. Depletion of PRR33 led to aggregation of DESMIN (Figs. 7A, B; S11A, B, white arrows) and disordered distribution of mitochondria in myofibers (Fig. 7C, D). These observations suggest the possibility that PRR33 is required for the binding of mitochondria to DESMIN. To evaluate the impact of *Prr33* inhibition on mitochondrial organization and DESMIN distribution, we co-labeled both mitochondria (by anti-TOMM20 antibody) and DESMIN (by anti-DESMIN antibody) in the cells. In undifferentiated myoblasts cultured in growth medium (GM), DESMIN staining was very dim and barely detectable, and *Prr33* inhibition did not visibly affect the intracellular pattern of mitochondria (Fig. S12A). However, in formed myotubes, *Prr33* deficiency resulted in abnormal distribution of DESMIN and mitochondria (Asterisk (*): accumulated DESMIN; White arrows: disorganized mitochondria.) (Fig. 7E, F). To verify the observation in vivo, we examined the intracellular distribution patterns of DESMIN, TOMM20 and PRR33 in muscle fibers using cryosection immunofluorescence staining. As illustrated in Fig. S12B–D, DESMIN, TOMM20, and PRR33 displayed co-localization. Notably, increased punctate or aggregated DESMIN and TOMM20 structures were observed in *Prr33* knockout muscle cryosections, suggesting that *Prr33* deficiency leads to abnormal DESMIN and mitochondrial distribution within myofibers (Fig. S12B–F).

To investigate the impact of PRR33 depletion on the association of mitochondria with DESMIN, we utilized the proximal labeling approach to assess the DESMIN-mitochondria interaction in C2C12 cells. As depicted in Fig. S12H, the mitochondrial protein TUFM was biotin-labeled in C2C12 cells by the DESMIN-Turbo-ID (Fig. S12G), reaffirming the proximity and interaction of DESMIN with mitochondria [49]. Remarkably, treatment with *Prr33*-gRNA substantially diminished the biotin labeling of the mitochondrial resident, suggesting a dependency of DESMIN-mitochondria association on PRR33. Overall, PRR33 emerges as a key regulator of DESMIN-mitochondrial interaction and their subcellular patterning in myocytes.

## Discussion

**Fig. 8:**
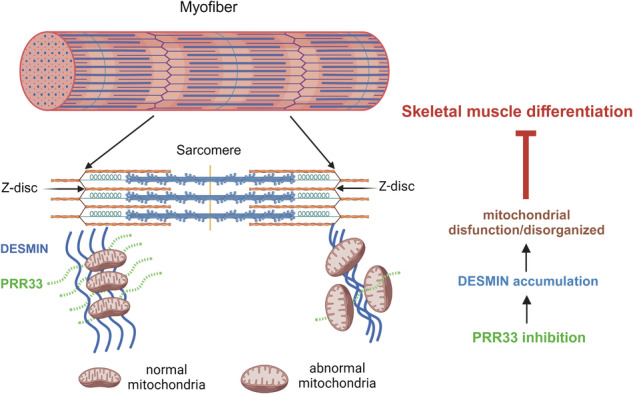
PRR33 regulates muscle differentiation by adapting DESMIN-mitochondria patterning to myogenesis. Schematic model that PRR33 interacts with DESMIN, regulates mitochondrial morphology, patterning and function, and modulates muscle differentiation. PRR33 deletion results in DESMIN accumulation, disordered mitochondrial morphology and impaired metabolic function, and eventually represses myoblast differentiate into myotube.

Mitochondrial content, morphology and distribution in myocytes reflect cellular metabolic states and are critical determinants of myogenesis and skeletal muscle function/performance. While the pivotal roles of these organelles in muscle biology and physiology are well recognized, the mechanisms that regulate mitochondrial organization in muscle cells remain poorly characterized [57]. The factors involved in this regulatory process in muscle cells are largely unknown. In this study, we uncovered that PRR33 binds to DESMIN, an intermediate filament protein crucial for mitochondrial transport and localization. Depletion of PRR33 in myocytes leads to DESMIN accumulation, resulting in mitochondrial disorganization and dysfunction, impeded differentiation, and impaired muscle performance. Our findings unveil a previously unrecognized model, wherein the pro-myogenic factor PRR33 interacts IF protein DESMIN, facilitating intracellular mitochondrial patterning in muscle cells and promoting myocyte differentiation (Fig. 8). This discovery offers new insights into the mechanisms governing mitochondrial networks in skeletal muscle cells.

During differentiation, myoblasts are exposed to a shift of energy demands. Alterations in mitochondrial content and organization occur to adapt myoblasts to these metabolic changes [21]. This presents a unique challenge for the muscle cells in coordinating the mitochondrial re-patterning with myogenesis. To achieve this coordination, the activity or expression of the involved factors should presumably correlate with myocyte differentiation. Indeed, in the C2C12 model, the mRNA level of PRR33 increases concurrently with myogenic processes, mirroring the pattern observed for DESMIN [59, 67], which is crucial for mitochondrial transport and localization in muscle differentiation. PRR33 also binds to DESMIN and modulates mitochondrial patterning in muscle cells. These findings suggest that PRR33 and DESMIN may serve as “relays” between upstream myogenic signals and mitochondrial patterning in the cells and facilitate the coordination of organelle reorganization with muscle differentiation.

DESMIN forms intermediate filaments extending from the Z-disk, linking adjacent sarcomeres to provide structural support and stability to myofibrils [58, 68]. It interacts with various Z-disk proteins, including PRR33, as demonstrated in our study. The DESMIN intermediate filament network offers mechanical support and contributes to sarcomere structure and the spatial organization of cellular components, such as mitochondria and the nucleus. In our investigation, deletion of *Prr33* resulted in a notable reduction in sarcomere length, a crucial outcome measure for muscle properties, and morphological abnormalities in mitochondria. These findings, together with observed dysregulation of the cytoskeleton and mitochondrial factors, aberrant DESMIN accumulation accompanied by respiratory function deficiencies in *Prr33* loss-of-function muscle cells, suggest that PRR33 plays a critical role in regulating the mechanical properties of the intermediate filament network and connections between the cytoskeleton and mitochondria in muscle cells. Notably, *Prr33* deletion did not significantly alter Z-disc lengths, indicating a milder impact on Z-disc morphology compared to DESMIN. Therefore, the involvement of multiple and additional factors in regulating DESMIN and Z-disc integrity cannot be discounted. Our study illustrates that PRR33 serves as a key regulator of DESMIN. Future investigations into PRR33 and other involved molecules will deepen our understanding of the mechanisms regulating cytoskeleton-mitochondria organization in muscle cells.

Our results obtained from loss-of-function and gain-of-function, in vitro and in vivo assays consistently show that PRR33 is a critical pro-myogenic regulator of myoblast differentiation. How does PRR33 regulate myogenesis? The regulatory role(s) of mitochondria in determining muscle cell fates has been characterized. Inhibition of mitochondria function through genetic or pharmacological manipulations causes defects in muscle differentiation [69–71]. Therefore, we hypothesize that the pro-myogenetic function of PRR33 may be attributed to its regulatory effects on mitochondria.

Intriguingly, depletion of PRR33 in myoblasts results in a decrease in mRNA level of MYOD and MYOGENIN, key transcription factors activating myogenic program. Ectopic expression of PRR33 upregulates MYOD and MYOGENIN, further suggesting that MYOD and MYOGENIN are intimately modulated by PRR33. The result indicates that PRR33 may promote muscle differentiation by enhancing the expression of MYOD and MYOGENIN. Notably, impairment of mitochondrial function in myoblasts, by chloramphenicol, FCCP or oligomycin, inhibits MYOGENIN expression [19, 21]. Conversely, activation of the organelle by expressing p43 T3 receptor increases MYOGENIN level [20]. These regulatory effects are similar to those observed in our PRR33 loss-of-function and gain-of-function assays. However, expression of MYOD, another myogenic transcription factor regulated by PRR33, appears not to be altered by the mitochondrial manipulations [19, 20]. This raises the possibility that, besides mitochondrial regulation, additional and diverse pathways may participate in mediating the pro-myogenic effects of PRR33. Further study on these cellular events and their cross-talk will offer more insights into the regulatory networks governing muscle differentiation.

Our high-throughput Turbo-ID-MS assay reveals that PRR33 is in close proximity to numerous cytoskeleton proteins. And as a binding partner of DESMIN, PRR33 likely contributes to the structural integrity of cytoskeleton. Yet, how does PRR33 as a cytoskeleton protein affect the mRNA levels of MYOD and MYOGENIN? DESMIN and cytoskeleton can act upstream and are required for normal expression of *MyoD*, *Myogenin* and *Myf5* [47, 59]. DESMIN has been proposed to interlink the sarcolemma with the nuclear envelop and activate gene transcription through nuclear matrix proteins [48, 58, 72, 73]. PRR33 may play a role in the cytoskeletal control of myogenic gene expression, given that abrogation of PRR33 causes mis-localization of DESMIN in cells. Thus, the interplay between PRR33 and DESMIN not only affects intracellular mitochondrial organization, but also may be intimately involved in gene regulation in muscle cells. This presents an intriguing avenue for further research to elucidate the molecular mechanisms involved.

*Prr33* is localized in the antisense DNA strand partially overlap with the genomic region of the *Tnnt3*. It displays a similar expression pattern to *Tnnt3* in muscle tissues. The co-expression is likely the consequence of bidirectional transcription from the shared *cis* elements, or transcription of *Prr33* from a nucleosome-free region located in 3’ portion of *Tnnt3* gene body, similar to other cases previously reported [74]. Antisense transcripts have been shown to modulate the expression of their corresponding sense genes [40]. However, depletion of *Prr33* did not influence the expression level of *Tnnt3* in muscle cells, nor did it affect the expression of slow- and fast-twitch gene markers. This study primarily focuses on the interaction between PRR33 and DESMIN, and their regulatory impact on myogenesis. No significant myofiber type-specific effects were observed concerning the expression of muscle differentiation and myogenic markers (Fig. S6A–E). Nonetheless, the potential for PRR33 to exert fiber type-specific roles cannot be discounted. Future investigations in this topic would enhance our comprehension of this pivotal regulator in muscle biology.

DESMIN is essential for maintaining proper muscle structure and function. Mutations affecting DESMIN expression or bioactivity in human cause severe muscle disorders known as desmopathies. In this study, we identified PRR33 as a regulator of DESMIN in skeletal muscle. We speculate that the deregulation of their interplays may be associated with muscle diseases. The findings from our study could therefore stimulate new paradigm of research to develop new interventions to treat the defects.

## Materials and methods

### Mice and animal care

The mouse strain *Prr33*^*fl/fl*^ was procured from Nanjing Biomedical Research Institute of Nanjing University (NBRI, Nanjing, China), while *HSA-Cre* mice were obtained from the Jackson Laboratory (Bar Harbor, Maine, USA). Genotyping of the mice was performed using protocols provided by the suppliers. All mice used in the study had a C57BL/6 J genetic background. The study was conducted in accordance with the Public Health Service Guide for Care and Use of Laboratory Animals and was approved by Ethics Committee of the Second Affiliated Hospital of Zhejiang University School of Medicine. The mice were kept on a 12 h light/dark cycle, fed a germ-free diet, and given access to fresh sterilized water. For post-operative pain management, the mice were given a single dose of 0.1 mg/kg Buprenorphine intraperitoneally and repeated doses of 0.1 mg/kg Buprenorphine subcutaneously every 8 h for a total of 3 days.

### Cell culture and maintenance

C2C12 cells were cultured in growth medium consisting of DMEM high glucose, 20% fetal bovine serum, 100 U/ml penicillin, and 100 mg/ml streptomycin. Once the density reached approximately 80%, the cells were shifted to differentiation medium (DMEM high glucose, 2% Horse Serum, 100 U/ml penicillin, and 100 mg/ml streptomycin), to initiate differentiation. The differentiation medium was replaced daily to ensure proper differentiation.

The HEK 293T cell line was obtained from the American Type Culture Collection (USA) and was cultured in RPMI-1640 medium supplemented with 10% (v/v) fetal bovine serum, 2 mM L-glutamine, 100 U/ml penicillin, and 100 mg/ml streptomycin.

### Primary myofiber Isolation and immunostaining

Myofibers were isolated from the TA muscle as previously described [35]. Fibers fixed in 4% paraformaldehyde and blocked by blocking solution (5% goat serum and 0.1% Triton-X-100 in PBS) for 1 h for immunostaining.

### Generation of CRISPR sgRNA lentivirus and infection of C2C12

We designed two guide RNAs to target the *Prr33* gene. These guide RNAs were cloned into the Lenti-CRISPR V2 vector, which was obtained from Addgene (No. 52961) [75]. Lenti-X 293 T cells (Clontech, Cat. No. 632180) were prepared in a 10 cm dish and transfected with 13.3 μg of the Lenti-CRISPR v2 plasmid, 7.5 μg of the psPAX2 plasmid, and 5 μg of the pMD2.G plasmid, using Lipofectamine 2000 (Invitrogen). The psPAX2 and pMD2.G vectors were obtained from Addgene (Nos. 12260 and 12259). The transfection was performed 12 h after preparing the cells. After two days, the culture medium containing the lentivirus was filtered through a 0.45 μm filter and concentrated using a Beckman ultracentrifugation machine. The virus was resuspended in 100 μl of PBS and stored at −80 °C until use. A list of gRNAs used for CRISPR/CAS9 in this study can be found in Table S1.

The desired amount of virus stock was used to infect C2C12 cells, with a target multiplicity of infection (MOI) of 5 for 2 days. Puromycin (Sigma, 2 ug/ml) was added to the culture medium to select positive cells for an additional 3 days. The genomic DNA of selected cells was purified using the Genomic DNA Purification Kit (Accurate Biotechnology). Finally, PCR genotyping was performed to confirm the deletion of the *Prr33* gene. A list of primers used for genotyping CRISPR/CAS9 knockout cells and mouse in this study can be found in Table S2. A list of shRNA and siRNAs in this study can be found in Table S3.

### Quantitative Real-Time PCR

The total RNA was extracted from muscle tissue or cell samples using TRIzol reagent (Life Technologies). To remove any residual genomic DNA, the RNA samples were treated with DNase I (Life Technologies). Intron-spanning primers were designed for the qPCR analysis. The qPCR signal was detected using the VII7 Real-time PCR System with SYBR Green qPCR Master Mix (Vazyme Biotech). Data normalization was performed using *18S-Rrna* or *Rpl19* signal as the reference. A list of primers used for qRT-PCR in this study can be found in Table S4.

### Western Blot analysis

The protein lysates were harvested by using RIPA buffer supplemented with protease inhibitors cocktail and 1 mM PMSF at 4 °C for 15 min. After determining the concentration of proteins in the lysates, samples were denatured by heating in a 95 °C-metal bath for 5 min and loaded onto SDS-PAGE gels for separation of proteins based on their molecular weight. The separated proteins were then transferred to PVDF membranes for detection. The membranes were then incubated with primary antibodies overnight at 4 °C, followed by three washes with TBST buffer (TBS with Tween-20) and incubation with HRP (horseradish peroxidase) conjugated secondary antibodies for 1 h at room temperature. Finally, protein bands were visualized by using ECL reagents and a Bio-Rad ChemiDoc imaging system. A detailed list of the antibodies used in this study can be found in Table S5.

### Grip strength measurement

Mice have the tendency to grasp a horizontal metal grid while being suspended by their tails. To measure the grip strength, 4-week-old mice were subjected to the grasping force measurement (Ugo Basile 47200). When the mice grasp a metal grid which was connected to a force transducer, they were gently pulled horizontally to produce a force until the grip was released. Top five values will be scored.

### Endurance training and fatigue experiments

An accelerating rotarod device (Harvard Apparatus Panlab; LE8205) was used to evaluate the motor coordination and balance. Mice were trained for 3 consecutive days before the experiment. The acceleration settings were 5, 15, or 30 rpm, starting from the lowest acceleration. The average latencies to fall from the rotating rod during the testing periods were calculated for each mouse. Three repeated trials were investigated on the day of testing.

### Histology and Immunostaining

The muscle samples were fixed in 4% paraformaldehyde overnight and embedded in paraffin using standard procedures. 5μm thick sections were then stained with hematoxylin and eosin (H&E) and analyzed under a microscope (Leica).

For immunofluorescence staining of paraffin-embedded muscle sections, samples fixed with 4% paraformaldehyde were initially incubated with 10% goat serum in PBS and 0.1% Triton X-100 for 1 h at room temperature. Subsequently, the sections and plates were incubated overnight at 4 °C with primary antibodies. The primary antibodies were visualized with Alexa Fluor antibodies (Invitrogen) at a dilution of 1:2000 for 1 h. Following this, the samples were mounted and sealed using Fluoromount-G (Electron Microscopy Sciences, Cat: #17984-25). The sections also underwent counterstaining with wheat germ agglutinin (WGA), specifically Alexa Fluor 594 conjugate WGA, at a 1:400 dilution (Invitrogen) to facilitate cross-section analysis. Imaging was performed using a Nikon A1 confocal microscope.

Immunofluorescence staining of cryosections was conducted following previously established protocols [46, 76]. Biopsy cryomolds were immersed in 2-methylbutane and cooled in liquid nitrogen until the 2-methylbutane started to solidify. The recipient muscle was transfer to biopsy cryomolds containing O.C.T. compound, oriented longitudinally to facilitate cross-sectional slicing from top to bottom. The bottom of the cryomold was placed atop the chilled 2-methylbutane until fully frozen, then submerged for 1 min. Subsequently, the cryomold was removed and positioned on dry ice. Frozen samples were stored at −80 °C. Sections, 10μm thick, were prepared for immunofluorescence staining. To examine the co-localization of DESMIN, mitochondria, and PRR33, fresh frozen tissue sections were stained using anti-DESMIN, anti-TOMM20, and anti-PRR33 antibodies. Primary anti-bodies were visualized with Alexa Fluor antibodies. The samples were mounted and sealed with Fluoromount-G (Electron Microscopy Sciences, Cat: #17984-25) (see Fig. S12B–D).

### Proximity labeling Turbo ID

PRR33-Turbo-ID was generated by fusing *Prr33* with TurboID sequence (Flag-TurboID plasmid ordered from Addgene, No. 124646). About 48 h after Ad-*Prr33*-TurboID infection, cells were incubated with Biotin (400 μM) for 15 min. The samples were harvested, and biotin-labeled proteins were recovered by streptavidin magnetic beads purification. The bound proteins were resolved by SDS-PAGE, and the gel lanes were excised and cut from top to bottom into small pieces and subjected to mass spectrometry analysis.

DESMIN -Turbo-ID was generated by fusing *Des* with TurboID sequence. Ctrl-gRNA and *Prr33*-gRNA-treated C2C12 myoblasts were transfected by Ad-Turbo- *Des* -flag or mock and cultured in DM for 2 days. The cells were then treated with biotin (400 μM) to label DESMIN “neighbor Protein”. The samples were harvested, and biotin-labeled proteins were recovered by streptavidin magnetic beads purification. The total samples lysate was analyzed by western blot with Streptavidin-HRP antibody. Biotin-labeled proteins were recovered by streptavidin magnetic beads purification. The obtained samples were analyzed by western blot for mitochondrial protein detection (TUFM).

### Bimolecular Fluorescent Complimentary, BiFC

The bimolecular fluorescence complementation (BiFC) method involves combining two proteins with affinity for each other to form a complete fluorescent protein. This technique is used to detect the presence of protein-protein interactions and to visualize their spatial location.

The DNA sequence encoding Venus fluorescent protein fragments were amplified from CSII-EF-MCS-IRES2-Venus-PGK1 plasmid (Cat#: SP-2446, ordered from BRICS) to generate PRR33-VN (Venus N-term), Anxa2-VC (Venus C-term), ZYX-VC, and DESMIN -VC BiFC constructs. About 24 h after transfection of the BiFC plasmids into 293 T cells using Lipo3000 (Invitrogen), fluorescence signal was examined under a microscope.

### Co-immunoprecipitation, Co-IP

After overexpressing *Prr33* using an adenovirus in C2C12 cells for 2 days, we washed the cells and harvested them using Buffer B (20 mM HEPES pH 7.9, 20% glycerol, 0.42 M KCl, 0.2 mM EDTA, 0.5 mM DTT, 0.1% NP40). The cell lysates were centrifuged at 12000 rpm at 4 °C for 10 min, and the supernatant was then incubated with magnetic beads that had been combined with an antibody (IgG or flag) overnight at 4 °C. The magnetic beads were then adsorbed onto a magnetic rack with the mixture of beads and cell lysis solution, and the supernatant was discarded. The magnetic beads were cleaned with Buffer D (20 mM HEPES pH 7.9, 20% glycerol, 0.15 M KCl, 0.2 mM EDTA, 0.5 mM DTT, 0.1% NP40) 3–5 times. Finally, the protein was eluted from the beads for Western blot analysis.

### Mitochondrial oxidative stress analysis in *Prr33*-gRNA-treated C2C12 and si-*Des* C2C12

Pre-differentiated C2C12 cells (Ctrl-gRNA-treated Myoblast, *Prr33*-gRNA-treated Myoblast) and post-differentiated (3 days) C2C12 cells (Ctrl-gRNA-treated Myotube, *Prr33*-gRNA-treated Myotube) were seeded in 96-well plates from Agilent’s Seahorse XF system at a specified density. Transfecting si-RNA (si-NC and si-*Des*) in C2C12 cells which were cultured in growth medium, and then transferred to differentiation medium for 3 days.

On the day of the experiment, the original cell medium was replaced with Agilent’s medium (consisting of 25 mM glucose, 2 mM glutamine, and 1 mM pyruvate) and the cells were incubated at 37 °C in an incubator without CO_2_ for 45 min. The test plate was pre-hydrated the day before and a specified concentration of the drugs (Oligomycin: 1 μM; FCCP: 3 μM; Rotenone + Antimycin A: 2 μM) were added to the pre-hydrated plate. The oxygen consumption rates (OCR) were normalized to the seeded cell numbers.

### Muscle injury with BaCl_2_

Barium chloride (BaCl_2_) is commonly used to induce muscle injury in experimental studies, particularly in skeletal muscle regeneration research [46]. BaCl_2_ was dissolved in sterile saline to a final concentration of 1.2%. In total, 50 μl of BaCl_2_ solution or saline was injected with a 27 G needle into one TA muscle of *Prr33*^*fl/fl*^ and *Prr33*^*KO*^ mice. Muscles were then harvested at different time points post injection.

### RNA sequencing and transcriptome analysis

RNA samples from *Prr33*-gRNA-treated and Ctrl-gRNA-treated C2C12 were prepared for RNA-seq by Novogene (Beijing, China). Briefly, total RNA was isolated using Trizol (Invitrogen) and used to generate sequencing libraries with the NEBNext UltraTM RNA Library Prep Kit for Illumina (NEB, USA). Library quality was assessed using the Qubit2.0 Fluorometer and Agilent 2100 bioanalyzer, and qRT-PCR was used to accurately quantify the effective concentration of the libraries. The different libraries were then sequenced using Illumina NovaSeq 6000. For analysis, raw data (raw reads) in fastq format were processed using in-house Perl scripts. Clean data (clean reads) were obtained by removing reads containing adapters, reads containing poly-N, and low-quality reads from raw data. The reference genome and gene model annotation files were downloaded directly from the genome website. The index of the reference genome was built using Hisat2 (v2.0.5) and paired-end clean reads were aligned to the reference genome using Hisat2 (v2.0.5). Differential expression analysis of two conditions/groups (two biological replicates per condition) was performed using the DESeq2 R package (1.20.0). The p-values were adjusted using the Benjamini and Hochberg method. Enrichment analysis of differentially expressed genes was implemented using the cluster Profiler R package (3.8.1). The hierarchical clustering heatmap was generated using the ggplot library.

### Statistical analysis

The general measurement data was presented as mean ± SEM and analyzed using GraphPad Prism8.0.2(263) software. Comparisons between two groups with normal distribution and homogeneous variance were performed using a two-tailed unpaired Student’s *t-test*. For comparisons between more than two groups, one-way ANOVA with Dunnett’s multiple comparisons test was used. Two-way ANOVA with Sidak’s test was used for analyzing the effect of multiple factors. Non-parametric Kruskal-Wallis (H) test was used for data with non-normal distribution or uneven variance. Pearson test was used for correlation analysis and results were presented as Pearson’s r or r^2^ values with 95% confidence intervals (CI). A p-value of less than 0.05 was considered statistically significant. *P < 0.05; **P < 0.01; ***P < 0.001; ****P < 0.0001.

## Supplementary information

Supplemental figure S1

Supplemental figure S2

Supplemental figure S3

Supplemental figure S4

Supplemental figure S5

Supplemental figure S6

Supplemental figure S7

Supplemental figure S8

Supplemental figure S9

Supplemental figure S10

Supplemental figure S11

Supplemental figure S12

uncropped western blot pictures

supplemental table 1

supplemental table 2

supplemental table 3

supplemental table 4

supplemental table 5

supplementary figure and table legends

## Data Availability

All relevant data related to this manuscript are available from the authors on reasonable request. The accession number for the RNA-sequencing data described in this study is GSE252039. Original uncropped western blots are provided in supplemental data.
