# Supplementary figures and images for: Adapting cytoskeleton-mitochondria patterning with myocyte differentiation by promyogenic PRR33
Source: Cell Death Differ. 2024 Aug 15;32(1):177–93. doi: 10.1038/s41418-024-01363-w (PMC11742405; doi:10.1038/s41418-024-01363-w)

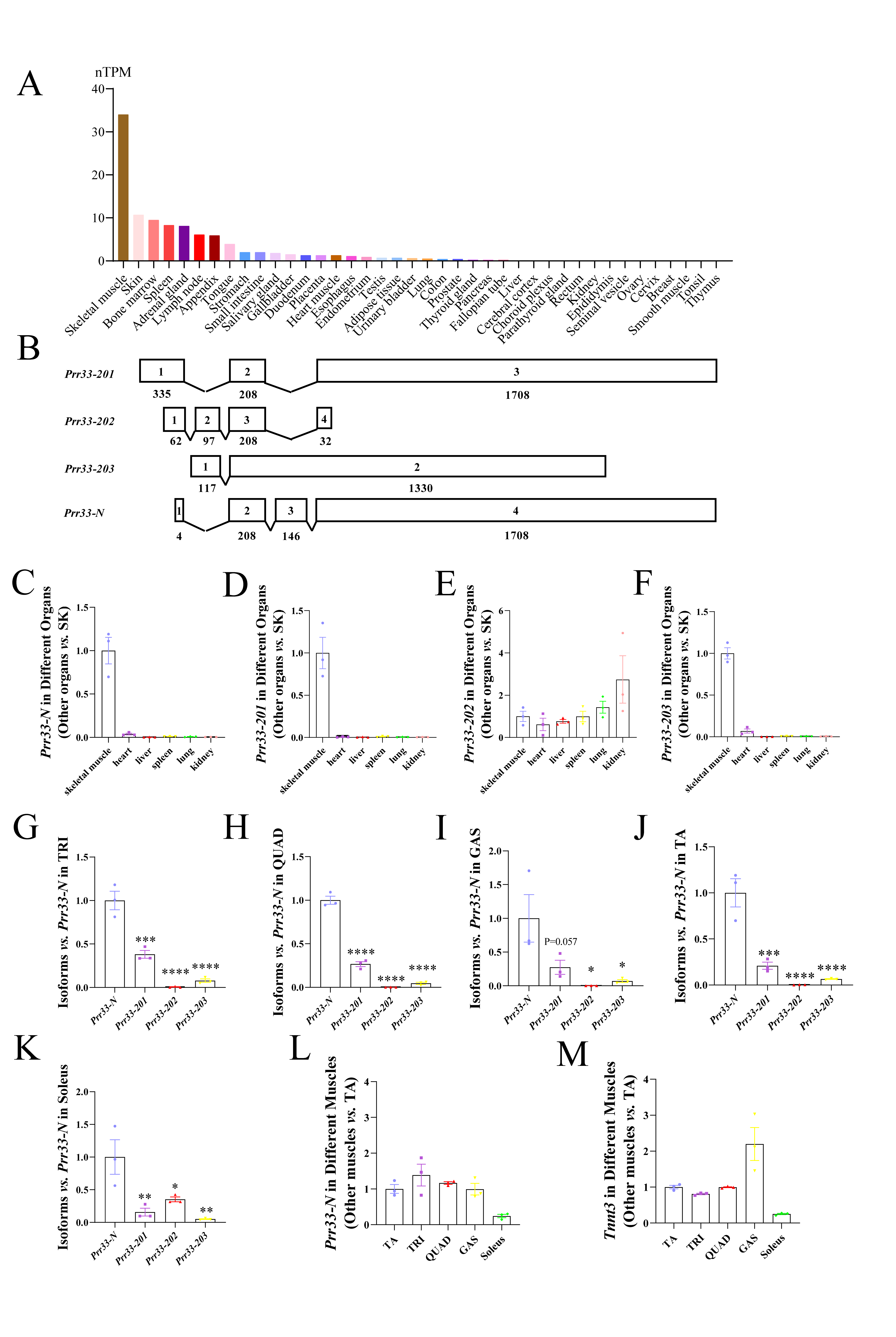

Supplement: Supplementary file 1 — Supplemental figure S1 [file 41418_2024_1363_MOESM1_ESM.tif]

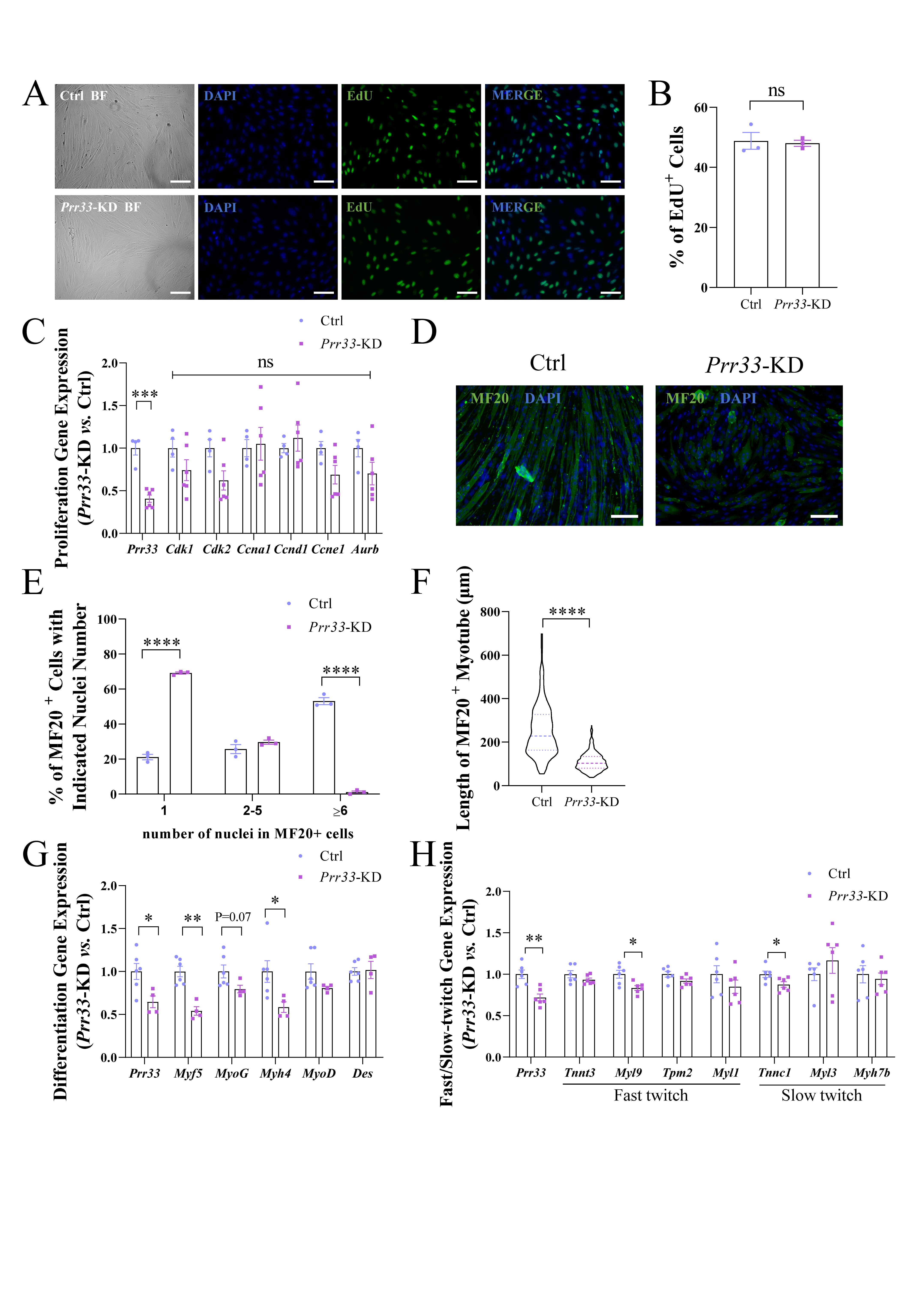

Supplement: Supplementary file 2 — Supplemental figure S2 [file 41418_2024_1363_MOESM2_ESM.tif]

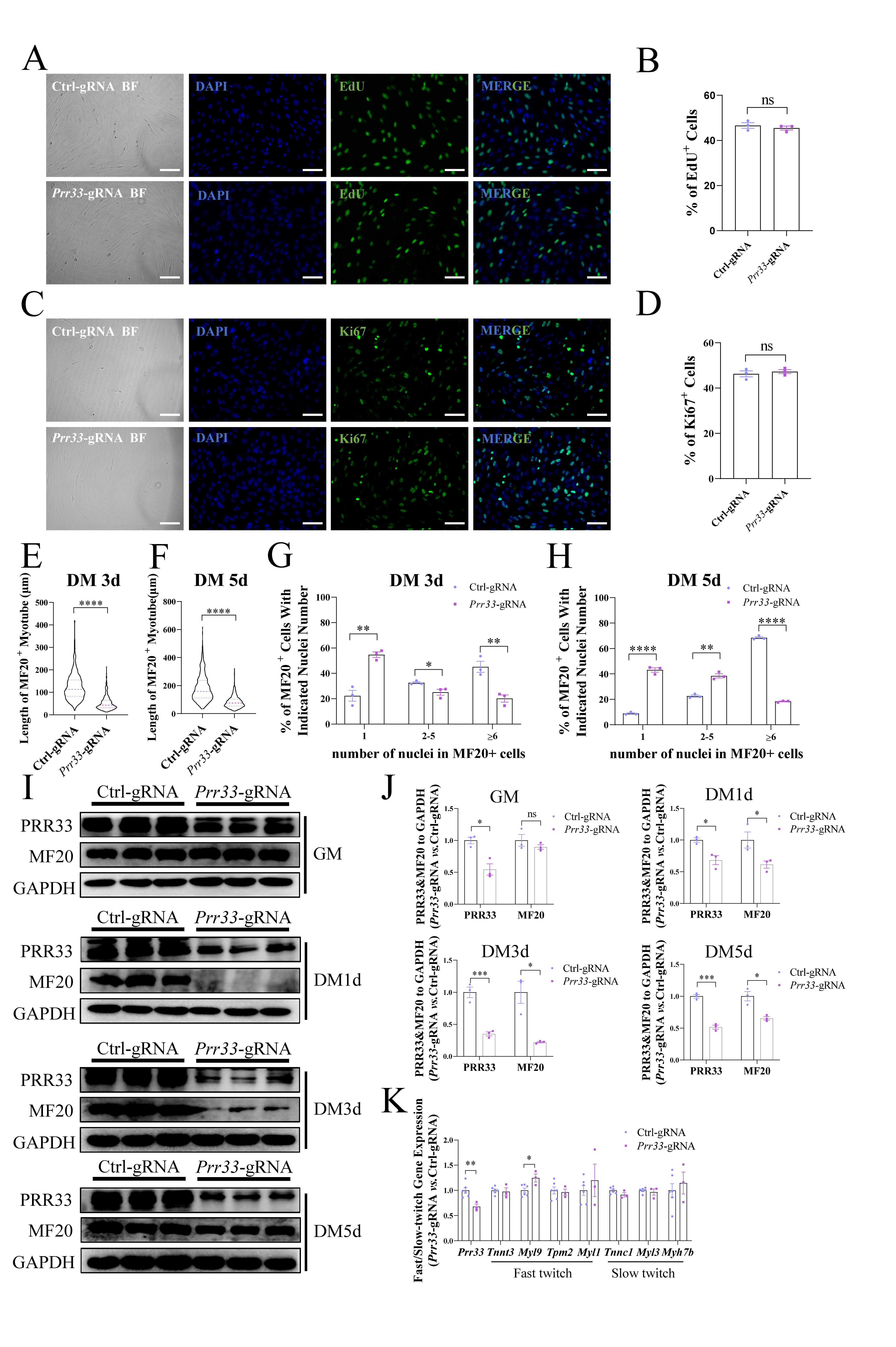

Supplement: Supplementary file 3 — Supplemental figure S3 [file 41418_2024_1363_MOESM3_ESM.tif]

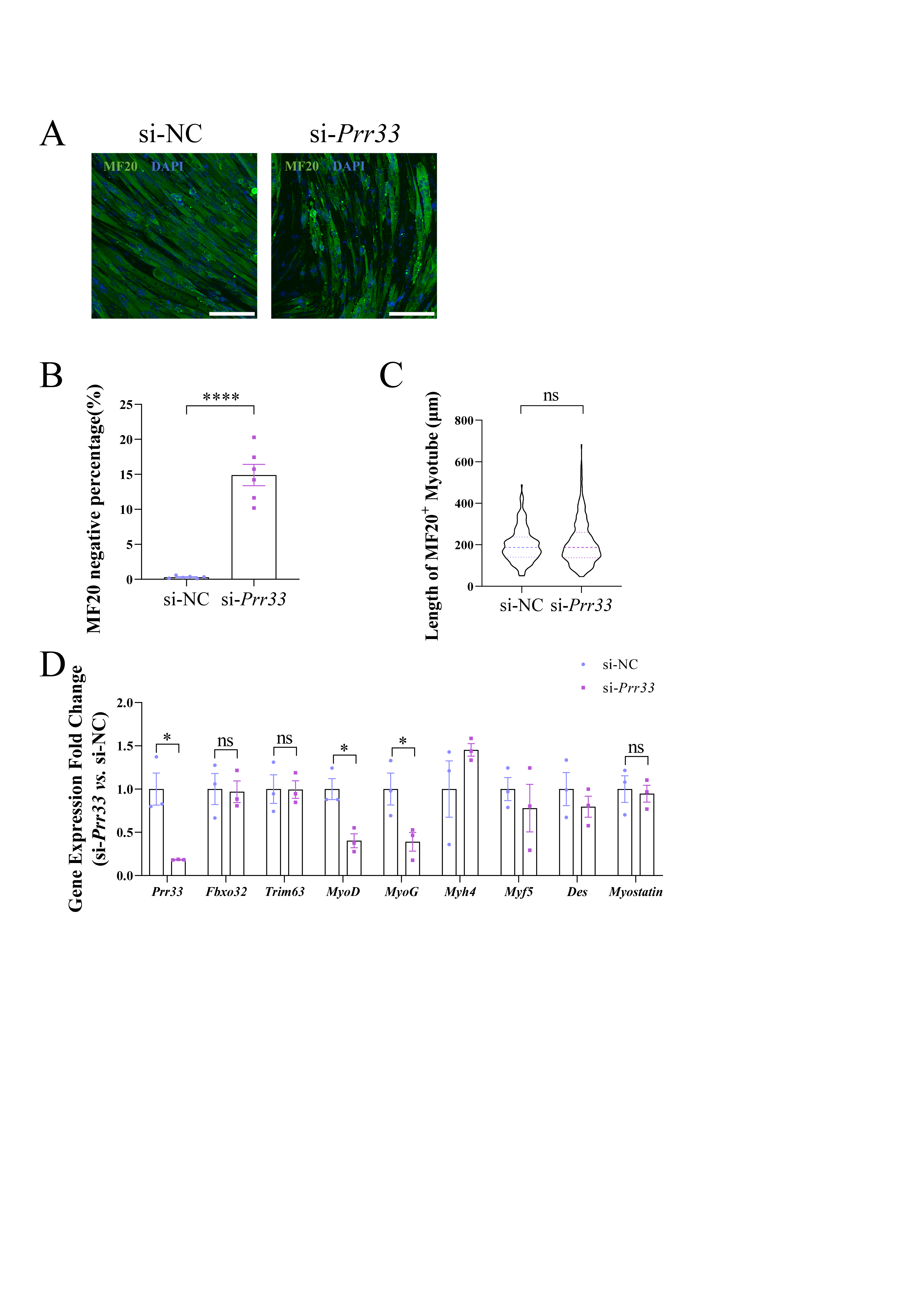

Supplement: Supplementary file 4 — Supplemental figure S4 [file 41418_2024_1363_MOESM4_ESM.tif]

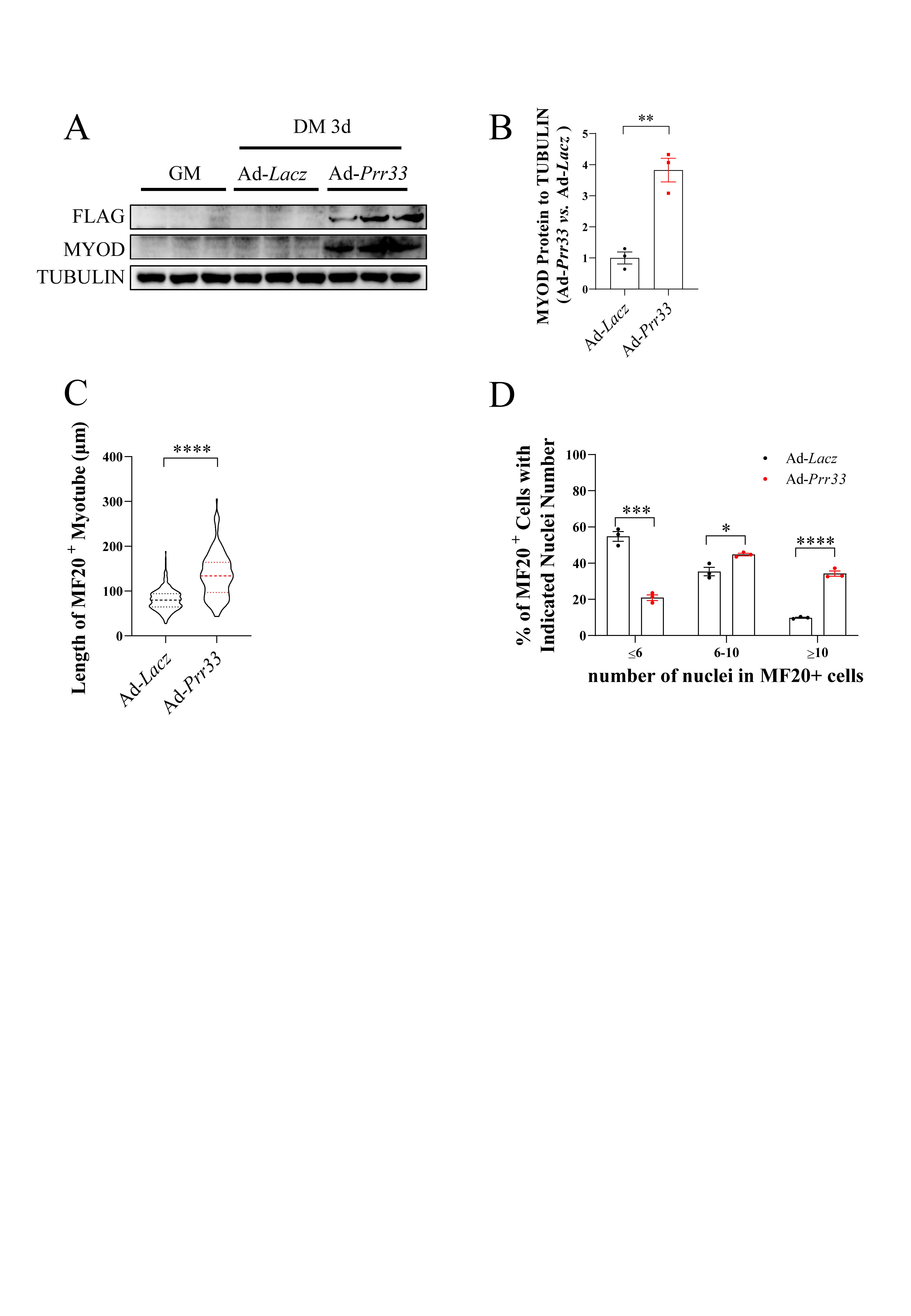

Supplement: Supplementary file 5 — Supplemental figure S5 [file 41418_2024_1363_MOESM5_ESM.tif]

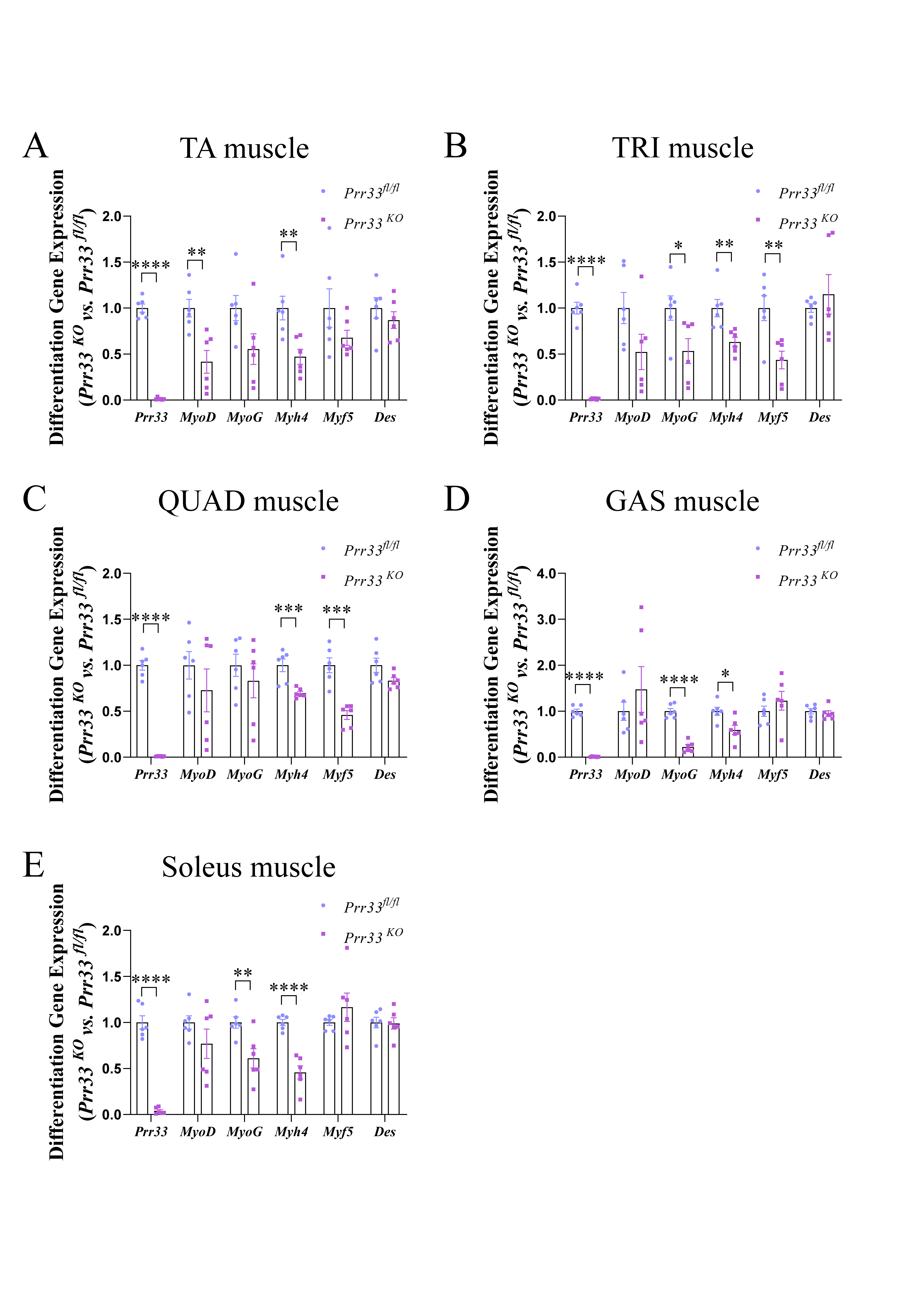

Supplement: Supplementary file 6 — Supplemental figure S6 [file 41418_2024_1363_MOESM6_ESM.tif]

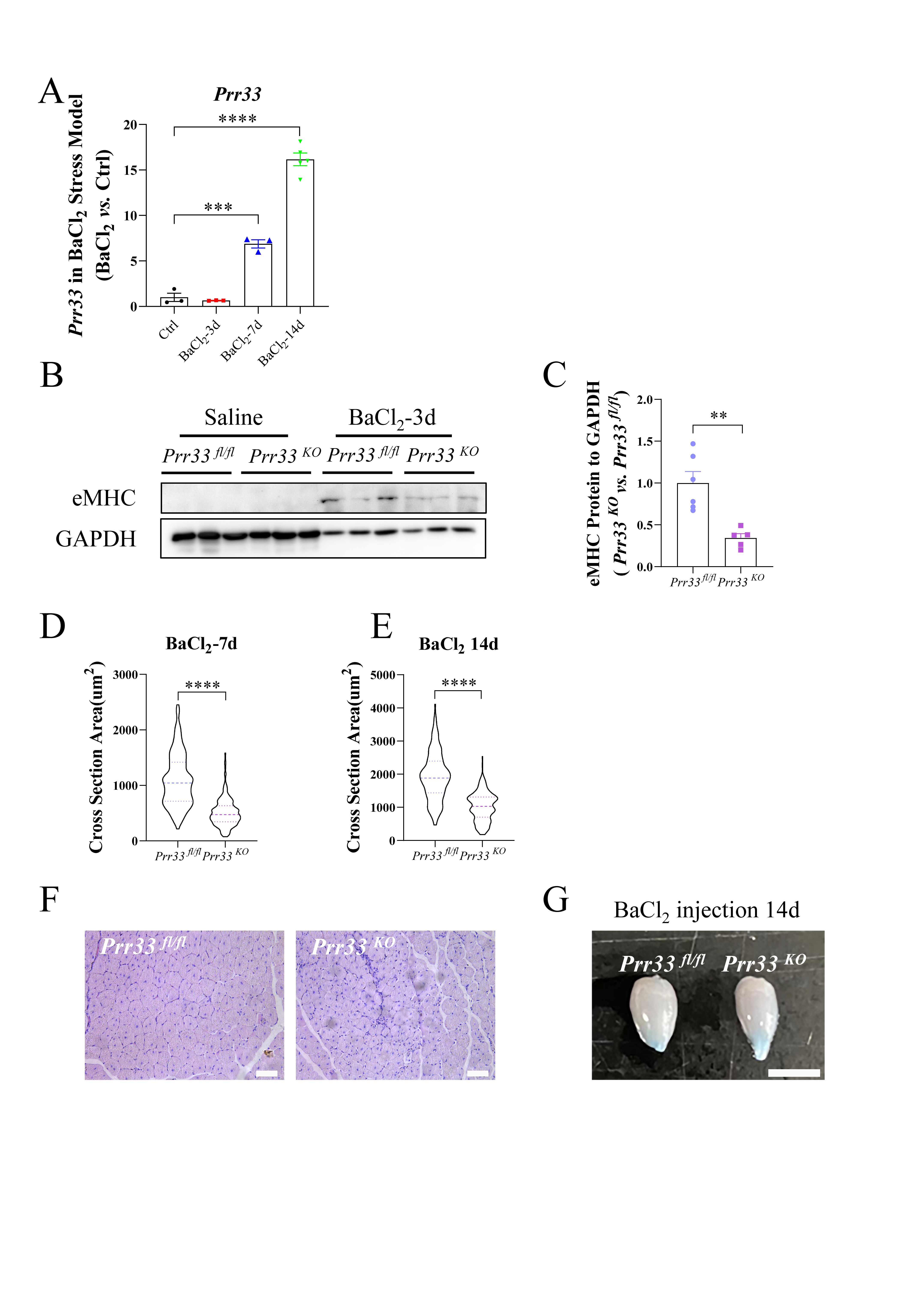

Supplement: Supplementary file 7 — Supplemental figure S7 [file 41418_2024_1363_MOESM7_ESM.tif]

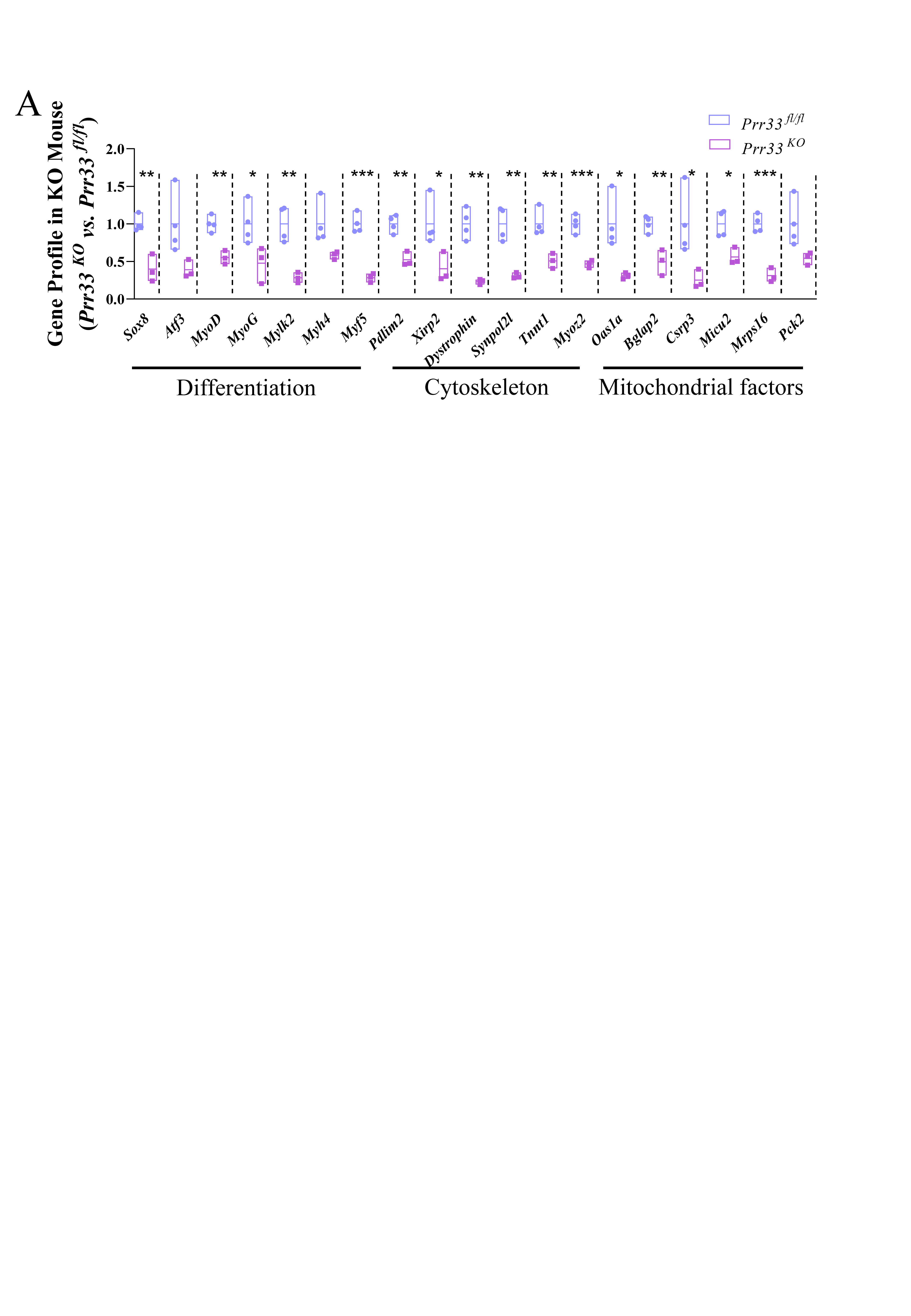

Supplement: Supplementary file 8 — Supplemental figure S8 [file 41418_2024_1363_MOESM8_ESM.tif]

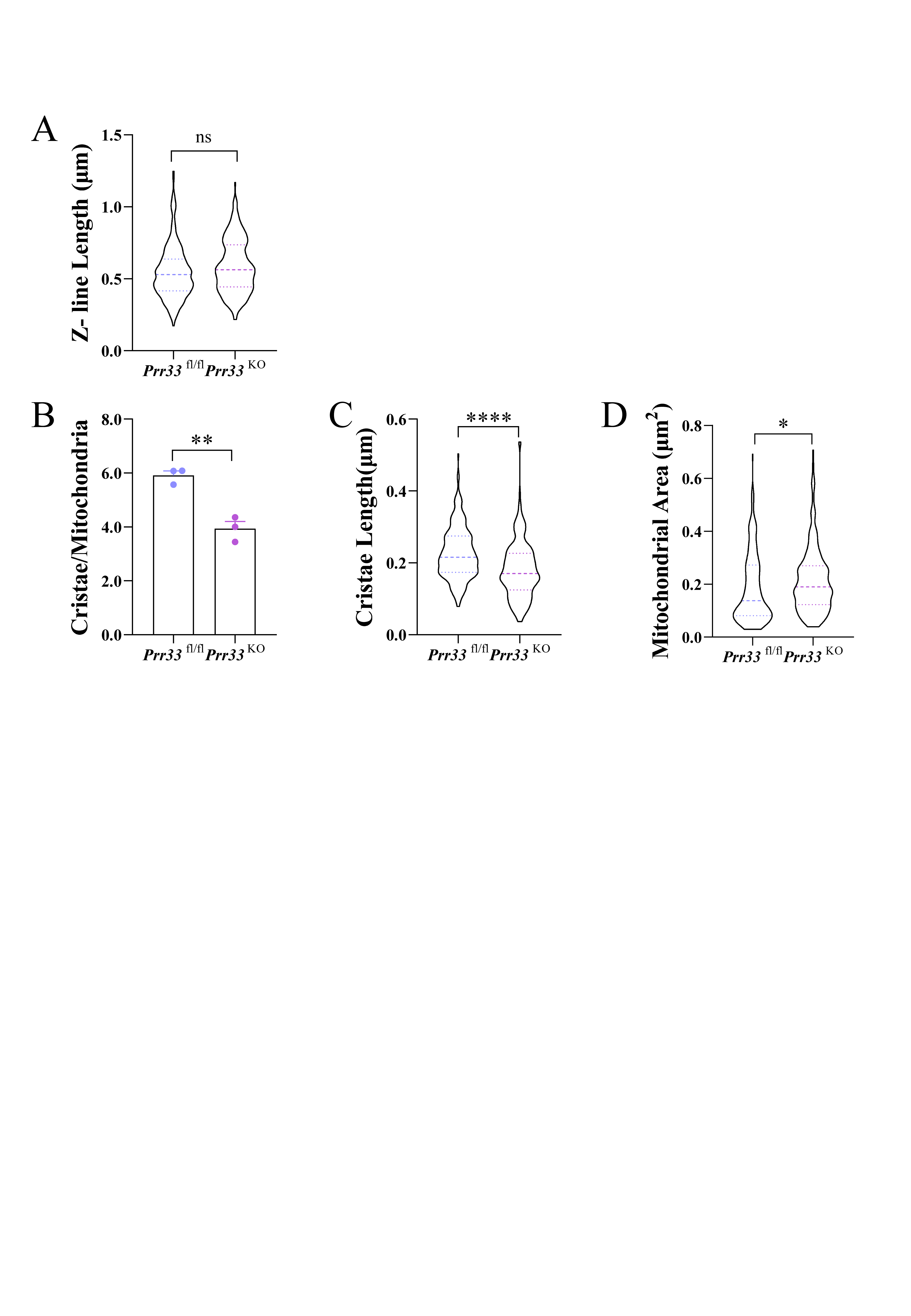

Supplement: Supplementary file 9 — Supplemental figure S9 [file 41418_2024_1363_MOESM9_ESM.tif]

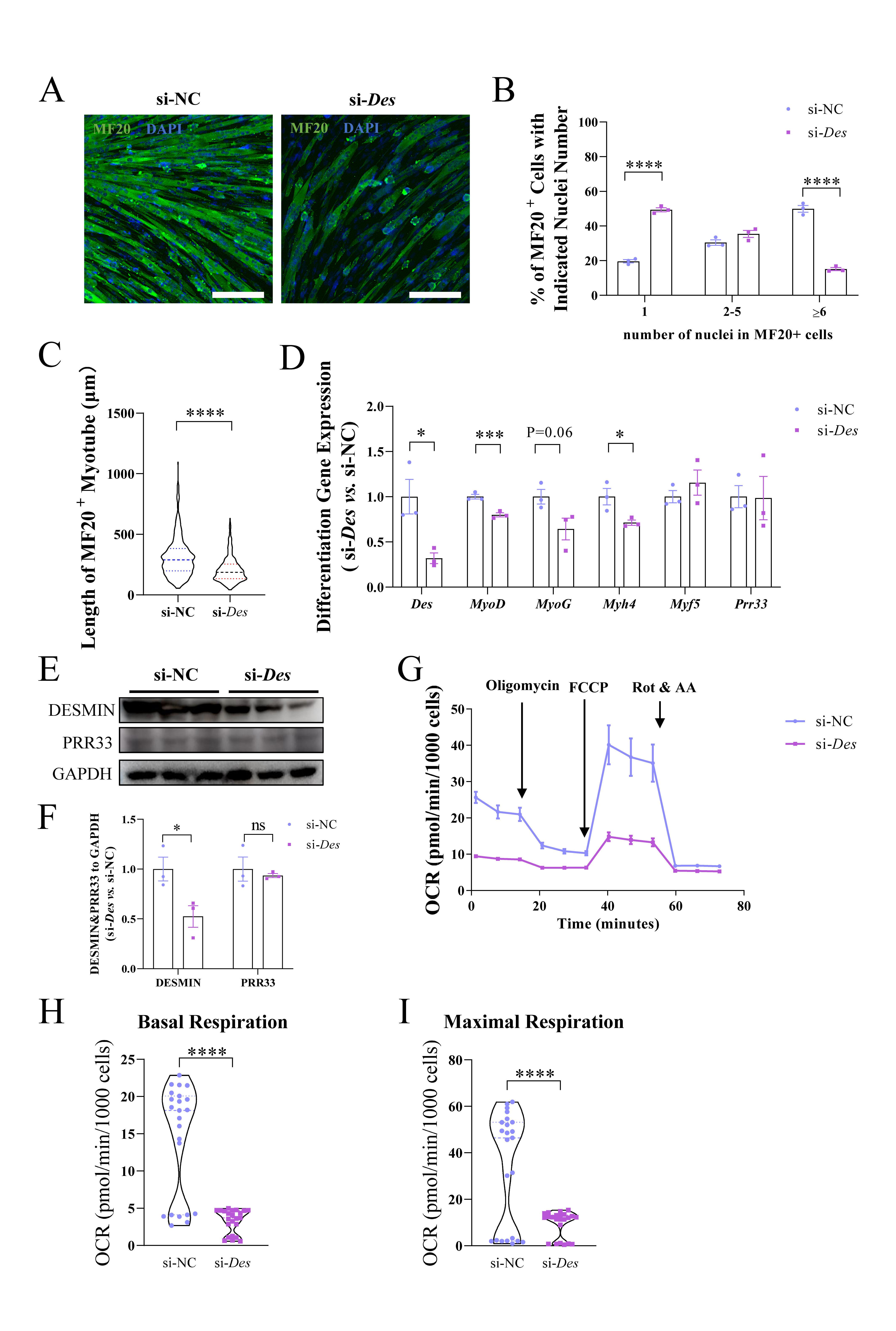

Supplement: Supplementary file 10 — Supplemental figure S10 [file 41418_2024_1363_MOESM10_ESM.tif]

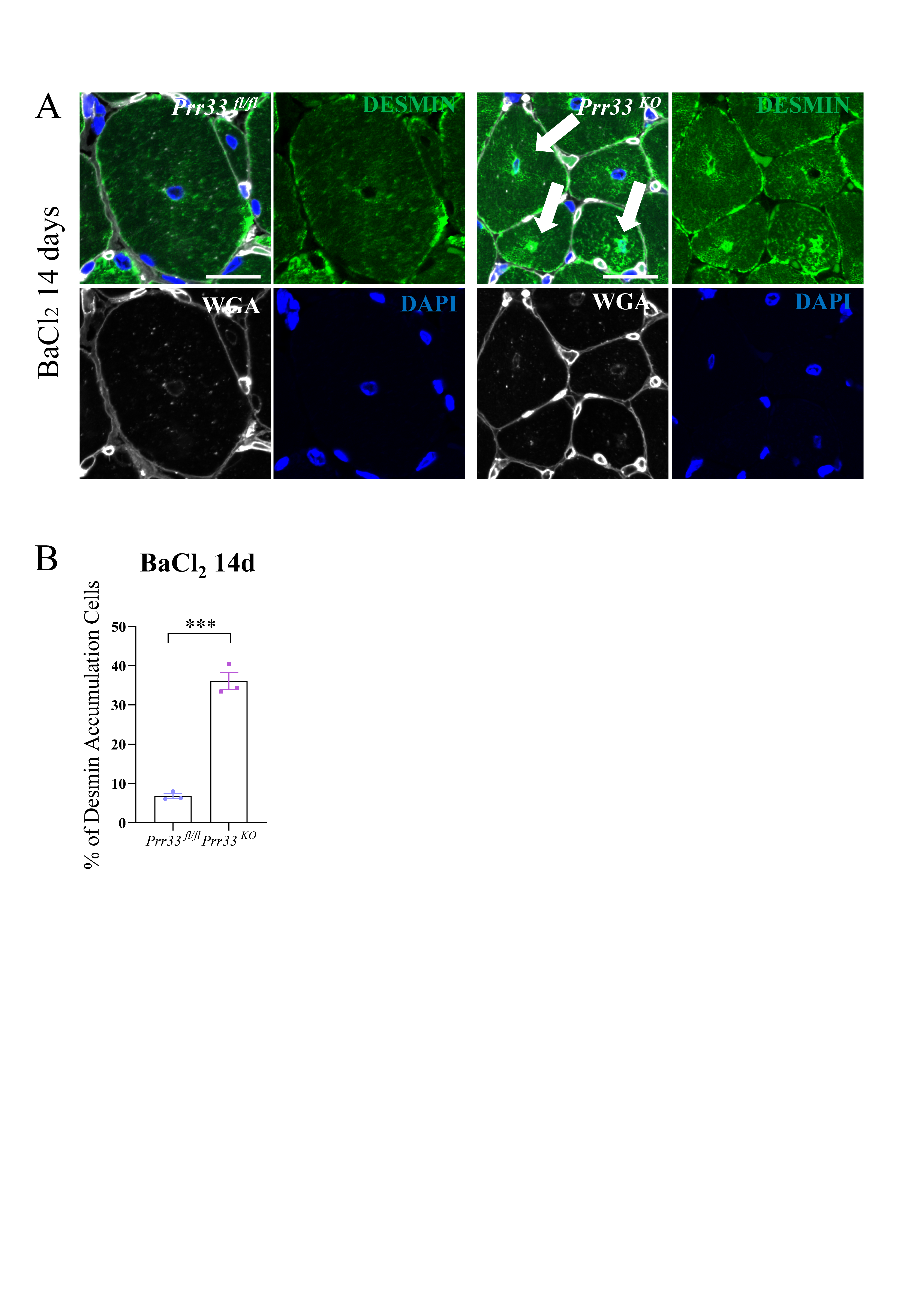

Supplement: Supplementary file 11 — Supplemental figure S11 [file 41418_2024_1363_MOESM11_ESM.tif]

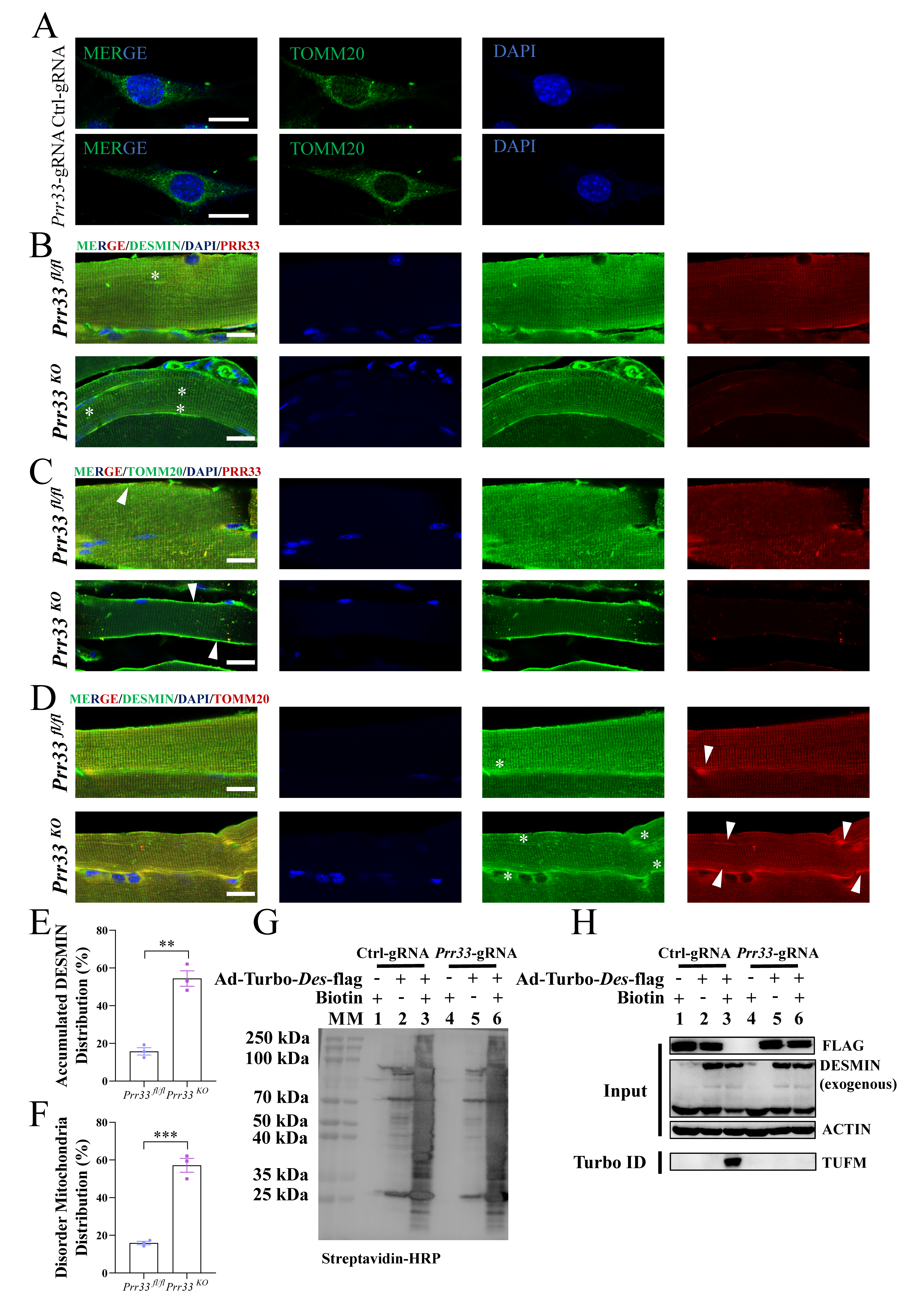

Supplement: Supplementary file 12 — Supplemental figure S12 [file 41418_2024_1363_MOESM12_ESM.tif]
