# Supplementary material for: Adapting cytoskeleton-mitochondria patterning with myocyte differentiation by promyogenic PRR33
Source: Cell Death Differ. 2024 Aug 15;32(1):177–93. doi: 10.1038/s41418-024-01363-w (PMC11742405; doi:10.1038/s41418-024-01363-w)
Supplement: Supplementary file 13 — uncropped western blot pictures [file 41418_2024_1363_MOESM13_ESM.pptx]

## Slide 1
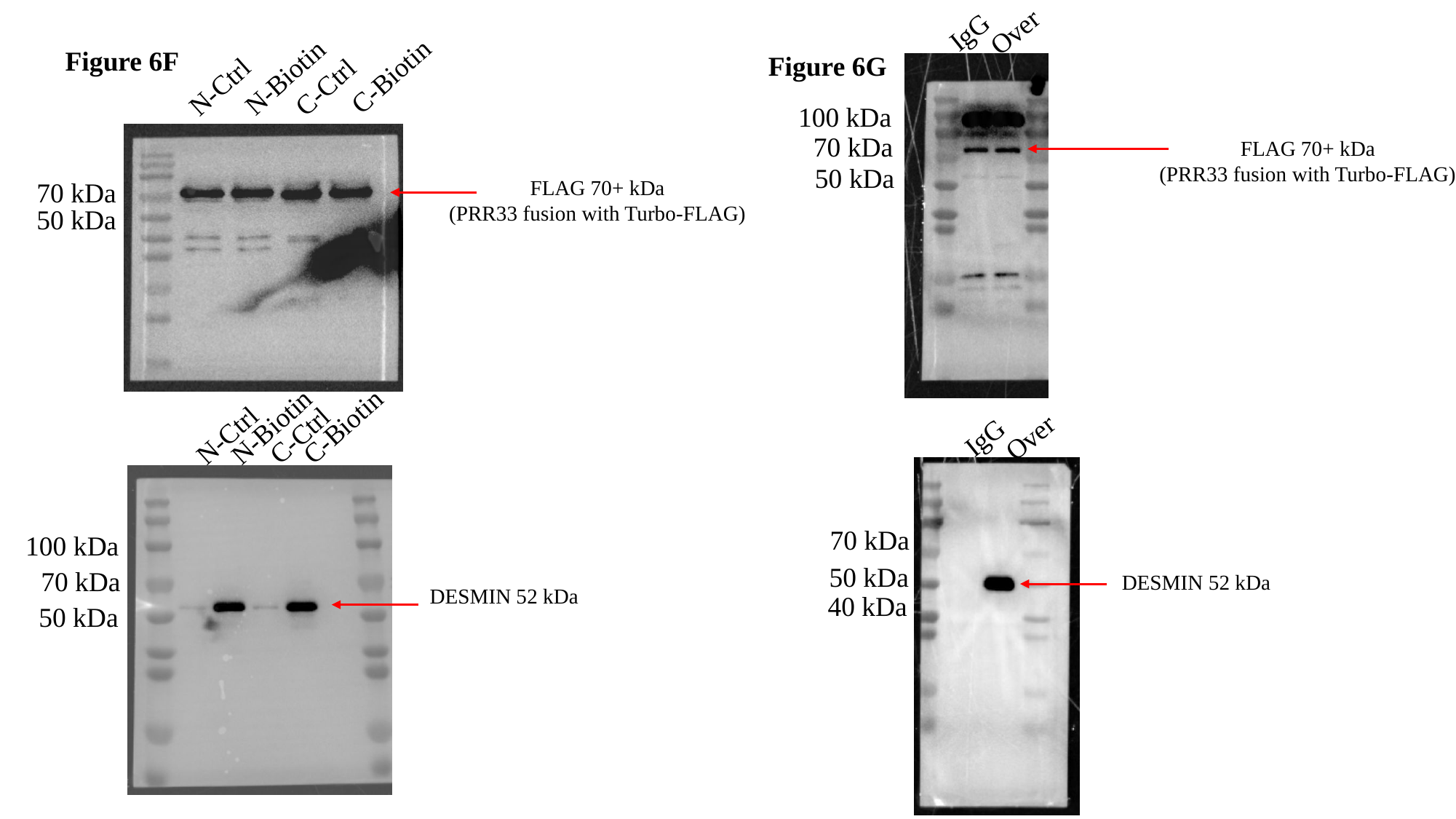

Over
IgG
Figure 6F
Figure 6G
C-Biotin
N-Biotin
N-Ctrl
C-Ctrl
100 kDa
70 kDa
FLAG 70+ kDa
(PRR33 fusion with Turbo-FLAG)
50 kDa
FLAG 70+ kDa
(PRR33 fusion with Turbo-FLAG)
70 kDa
50 kDa
N-Biotin
C-Biotin
N-Ctrl
C-Ctrl
Over
IgG
70 kDa
100 kDa
50 kDa
70 kDa
DESMIN 52 kDa
DESMIN 52 kDa
40 kDa
50 kDa

## Slide 2
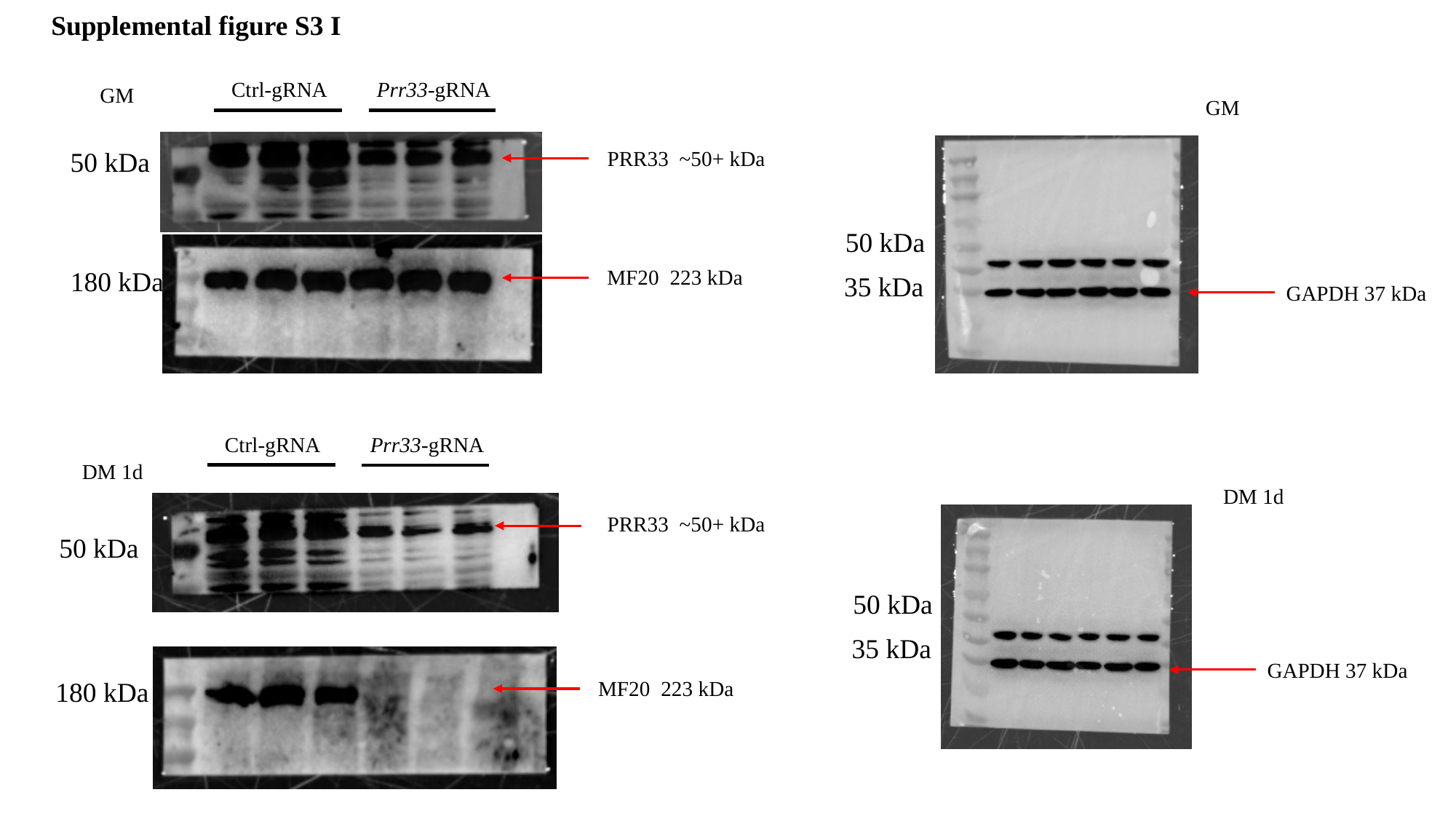

Supplemental figure S3 I
Ctrl-gRNA
Prr33-gRNA
GM
GM
50 kDa
PRR33 ~50+ kDa
50 kDa
180 kDa
MF20 223 kDa
35 kDa
GAPDH 37 kDa
Ctrl-gRNA
Prr33-gRNA
DM 1d
DM 1d
PRR33 ~50+ kDa
50 kDa
50 kDa
35 kDa
GAPDH 37 kDa
180 kDa
MF20 223 kDa

## Slide 3
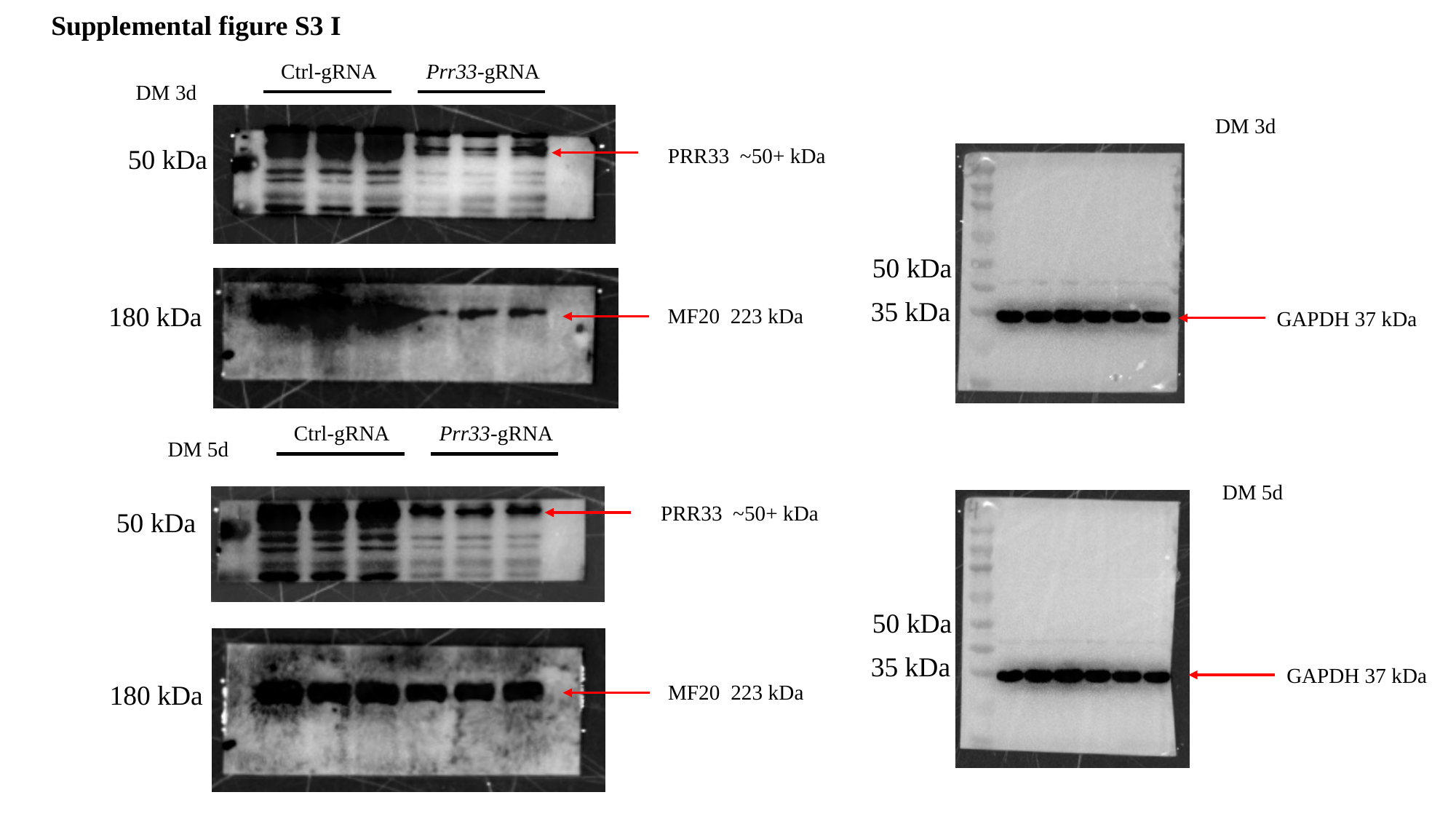

Supplemental figure S3 I
Ctrl-gRNA
Prr33-gRNA
DM 3d
DM 3d
50 kDa
PRR33 ~50+ kDa
50 kDa
35 kDa
180 kDa
MF20 223 kDa
GAPDH 37 kDa
Ctrl-gRNA
Prr33-gRNA
DM 5d
DM 5d
PRR33 ~50+ kDa
50 kDa
50 kDa
35 kDa
GAPDH 37 kDa
180 kDa
MF20 223 kDa

## Slide 4
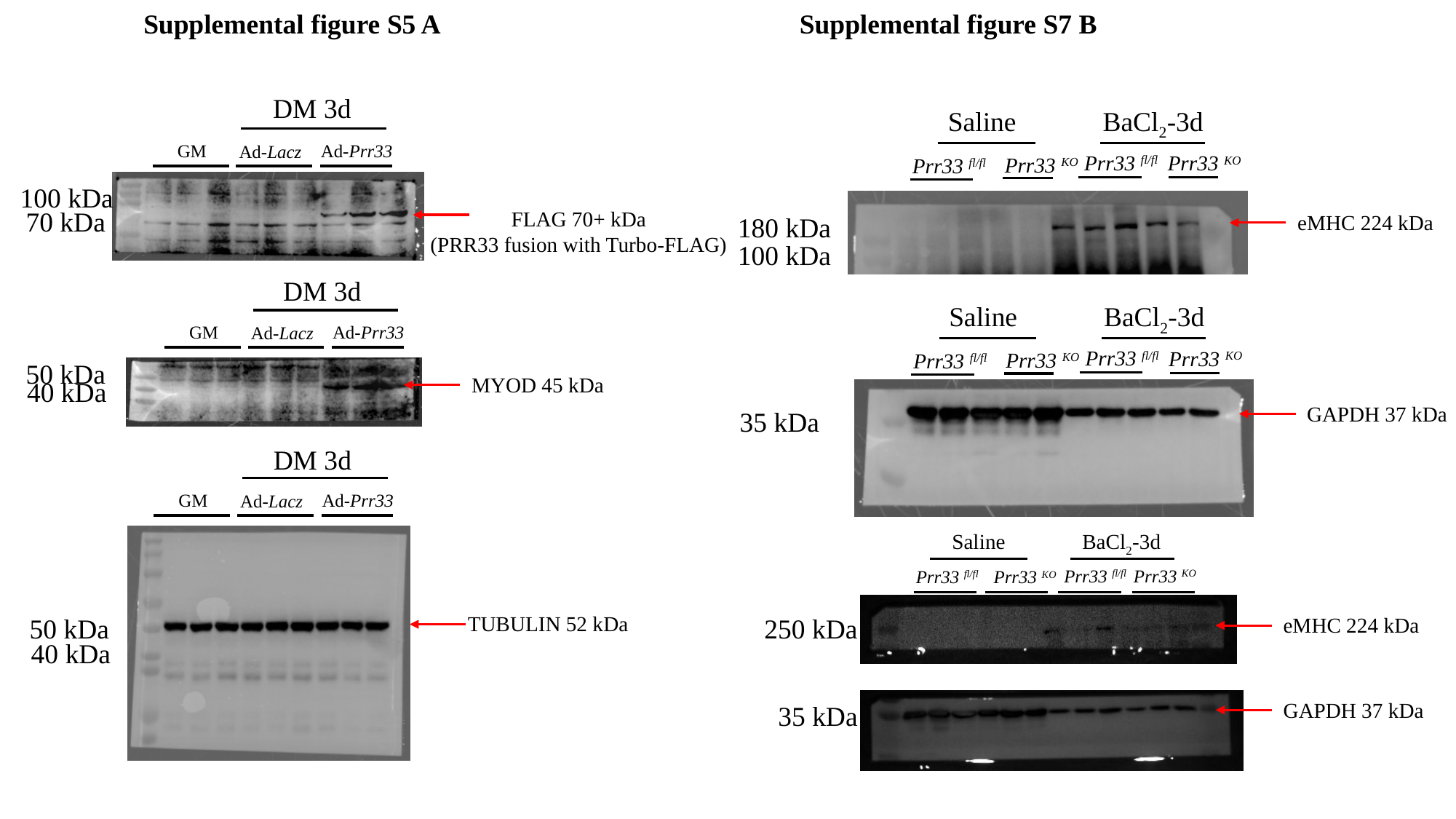

Supplemental figure S5 A
Supplemental figure S7 B
DM 3d
BaCl2-3d
Saline
Ad-Prr33
GM
Ad-Lacz
Prr33 fl/fl
Prr33 KO
Prr33 KO
Prr33 fl/fl
100 kDa
70 kDa
FLAG 70+ kDa
(PRR33 fusion with Turbo-FLAG)
eMHC 224 kDa
180 kDa
100 kDa
DM 3d
BaCl2-3d
Saline
Ad-Prr33
GM
Ad-Lacz
Prr33 fl/fl
Prr33 KO
Prr33 KO
Prr33 fl/fl
50 kDa
MYOD 45 kDa
40 kDa
GAPDH 37 kDa
35 kDa
DM 3d
Ad-Prr33
GM
Ad-Lacz
BaCl2-3d
Saline
Prr33 fl/fl
Prr33 KO
Prr33 fl/fl
Prr33 KO
TUBULIN 52 kDa
50 kDa
250 kDa
eMHC 224 kDa
40 kDa
GAPDH 37 kDa
35 kDa

## Slide 5
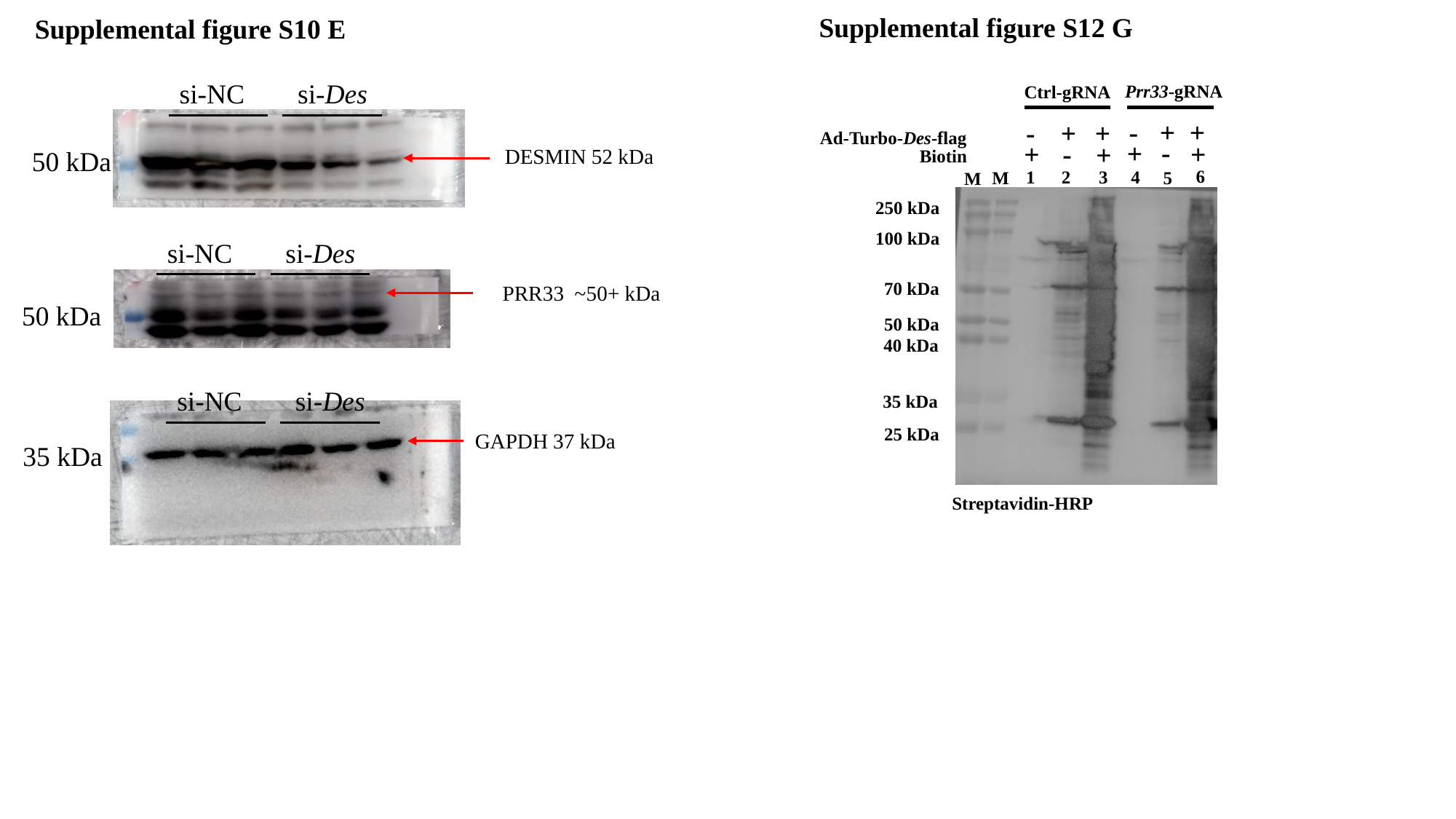

Supplemental figure S12 G
Supplemental figure S10 E
si-Des
si-NC
Prr33-gRNA
Ctrl-gRNA
-
+
+
-
+
+
Ad-Turbo-Des-flag
-
+
-
+
+
+
DESMIN 52 kDa
50 kDa
Biotin
6
4
1
3
2
M
5
M
250 kDa
100 kDa
si-Des
si-NC
70 kDa
PRR33 ~50+ kDa
50 kDa
50 kDa
40 kDa
si-Des
si-NC
35 kDa
25 kDa
GAPDH 37 kDa
35 kDa
Streptavidin-HRP

## Slide 6
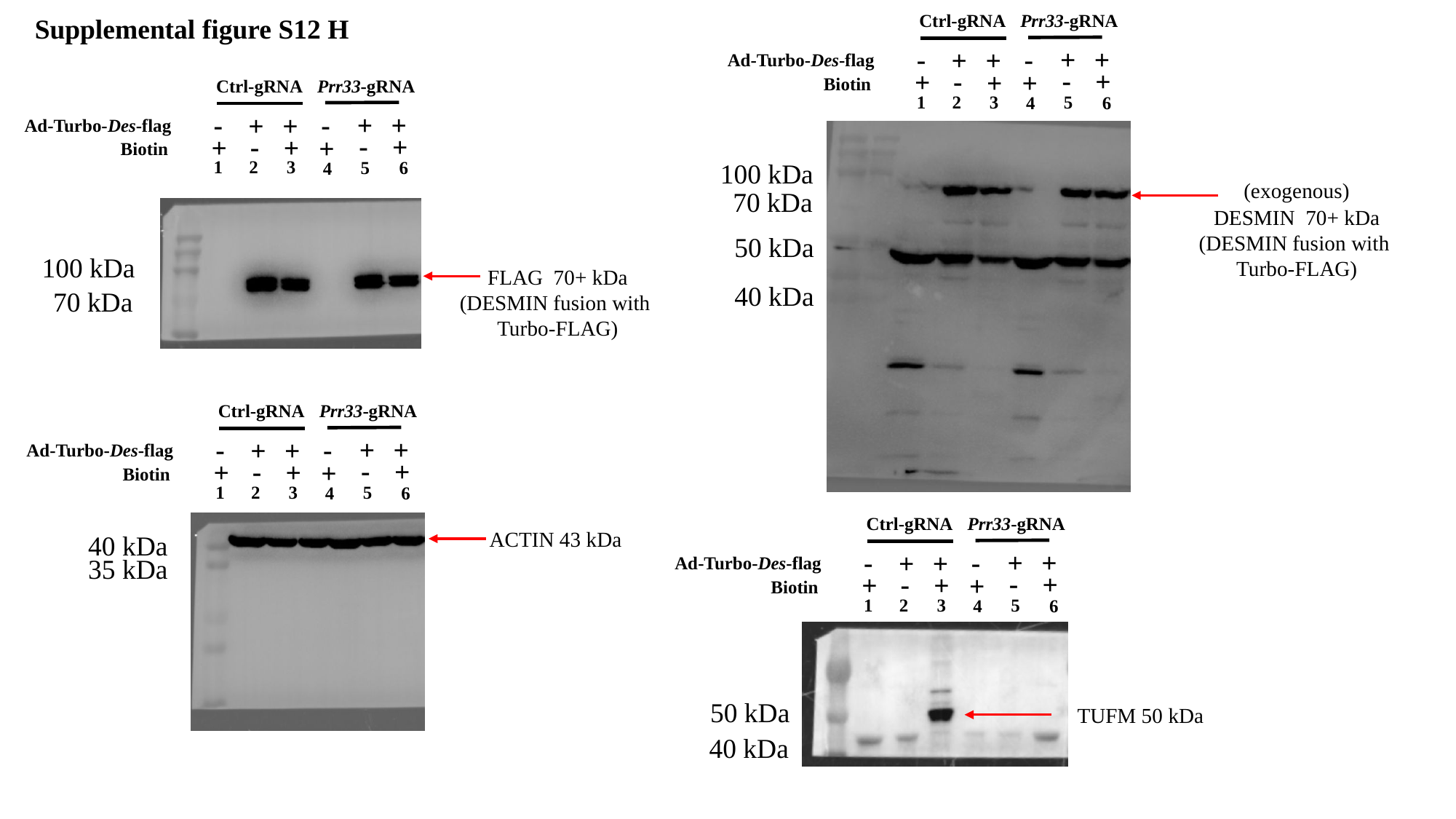

Ctrl-gRNA
Prr33-gRNA
Supplemental figure S12 H
-
-
+
+
+
+
Ad-Turbo-Des-flag
-
-
+
+
+
+
Biotin
Ctrl-gRNA
Prr33-gRNA
3
2
1
5
6
4
-
-
+
+
+
+
Ad-Turbo-Des-flag
-
-
+
+
+
+
Biotin
3
2
1
5
6
4
100 kDa
(exogenous)
70 kDa
DESMIN 70+ kDa
(DESMIN fusion with
Turbo-FLAG)
50 kDa
100 kDa
FLAG 70+ kDa
(DESMIN fusion with
Turbo-FLAG)
40 kDa
70 kDa
Ctrl-gRNA
Prr33-gRNA
-
-
+
+
+
+
Ad-Turbo-Des-flag
-
-
+
+
+
+
Biotin
3
2
1
5
6
4
Ctrl-gRNA
Prr33-gRNA
ACTIN 43 kDa
40 kDa
-
-
+
+
+
+
Ad-Turbo-Des-flag
35 kDa
-
-
+
+
+
+
Biotin
3
2
1
5
6
4
50 kDa
TUFM 50 kDa
40 kDa

## Slide 7
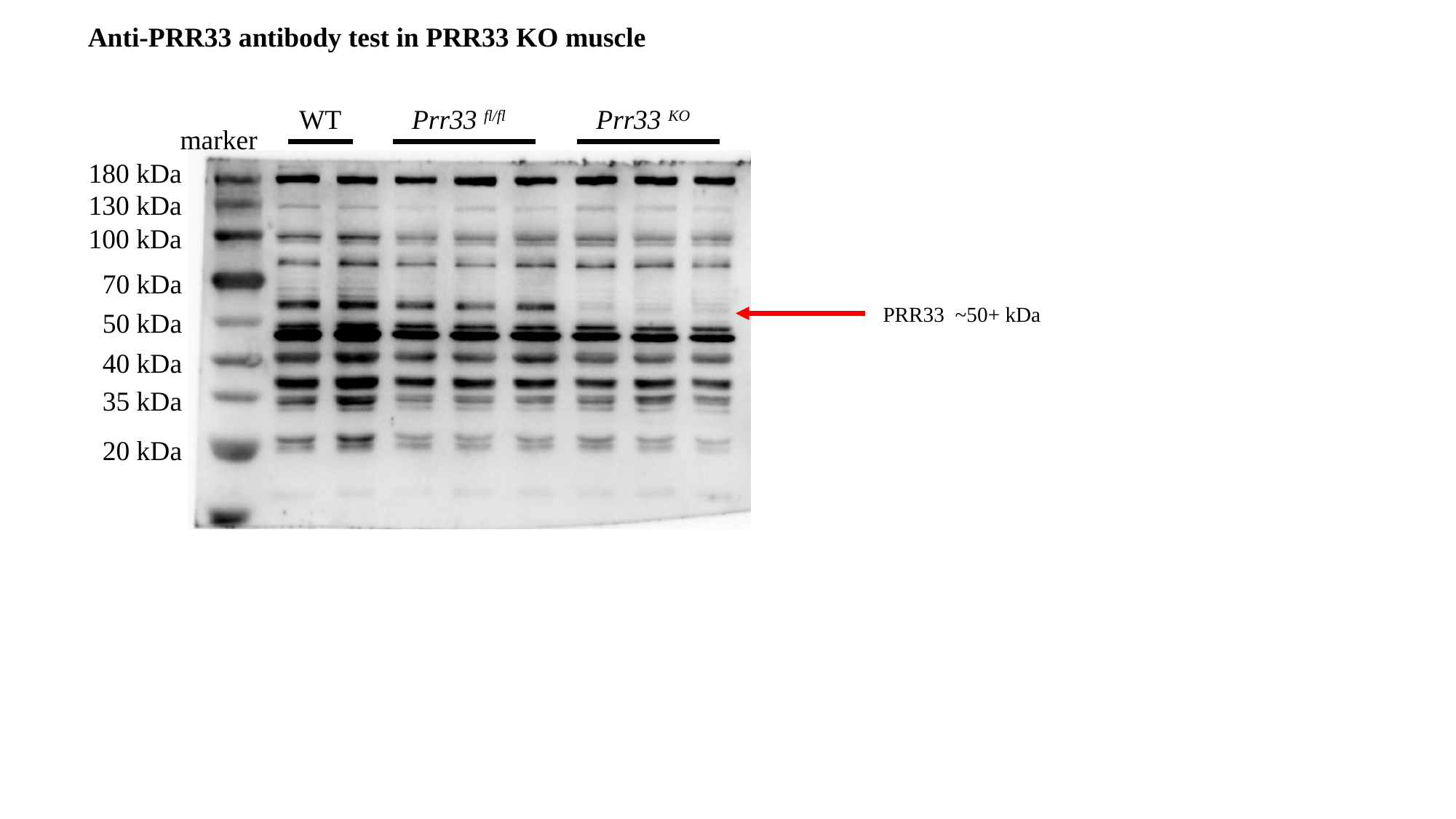

Anti-PRR33 antibody test in PRR33 KO muscle
WT
Prr33 fl/fl
Prr33 KO
marker
180 kDa
130 kDa
100 kDa
70 kDa
PRR33 ~50+ kDa
50 kDa
40 kDa
35 kDa
20 kDa
