# Supplementary material for: Adapting cytoskeleton-mitochondria patterning with myocyte differentiation by promyogenic PRR33
Source: Cell Death Differ. 2024 Aug 15;32(1):177–93. doi: 10.1038/s41418-024-01363-w (PMC11742405; doi:10.1038/s41418-024-01363-w)
Supplement: Supplementary file 19 — supplementary figure and table legends [file 41418_2024_1363_MOESM19_ESM.docx]

**Supplemental figure legends**

**Supplemental figure S1.** ***Prr33* is enriched in heart and skeletal muscle and is conserved in mammals**

(A) *Prr33* expression levels in different human tissues. The graph showed highest *Prr33* expression in skeletal muscle. Data was derived from the Human Protein ATLAS, HPA dataset. (B) Schematic for mouse *Prr33* genomic locus and mRNA isoforms (*Prr33-201*, *Prr33-202*, *Prr33-203* and *Prr33-N*). (C)The steady-state level of *Prr33-N* mRNA in a broad range of mouse tissues was assayed by qRT-PCR. The graph showed highest *Prr33-N* expression in striated muscle. N=3 for each group. (D) The steady-state level of *Prr33-201* mRNA in a broad range of mouse tissues was assayed by qRT-PCR. The graph showed highest *Prr33-201* expression in striated muscle. N=3 for each group. (E) The steady-state level of *Prr33-202* mRNA in a broad range of mouse tissues was assayed by qRT-PCR. N=3 for each group. (F) The steady-state level of *Prr33-203* mRNA in a broad range of mouse tissues was assayed by qRT-PCR. The graph showed highest *Prr33-203* expression in striated muscle. N=3 for each group. (G) The expression of *Prr33* isoforms in TRI muscle. The graph showed *Prr33-N* was the highest abundance isoform in TRI muscle. N=3 for each group. The data was presented as means ± SEM (One-way ANOVA). (H) The expression of *Prr33* isoforms in QUAD muscle. The graph showed *Prr33-N* was the highest abundance isoform in QUAD muscle. N=3 for each group. The data was presented as means ± SEM (One-way ANOVA). (I) The expression of *Prr33* isoforms in GAS muscle. The graph showed *Prr33-N* was the highest abundance isoform in GAS muscle. N=3 for each group. The data was presented as means ± SEM (One-way ANOVA). (J) The expression of *Prr33* isoforms in TA muscle. The graph showed *Prr33-N* was the highest abundance isoform in TA muscle. N=3 for each group. The data was presented as means ± SEM (One-way ANOVA). (K) The expression of *Prr33* isoforms in Soleus muscle. The graph showed *Prr33-N* was the highest abundance isoform in Soleus muscle. N=3 for each group. The data was presented as means ± SEM (One-way ANOVA). (L) The expression of *Prr33-N* in different kind of muscles (TRI, QUAD, GAS, TA and Soleus). N=3 for each group. (M) The expression of *Tnnt3* in different kind of muscles (TRI, QUAD, GAS, TA and Soleus). N=3 for each group.

**Supplemental figure S2. *Prr33* knock-down by lentivirus inhibits myoblast differentiation but has no influence on proliferation**

(A) C2C12 myoblasts transfected with lenti-sh*prr33* targeting *Prr33* gene and lenti-scramble were cultured in GM, fixed, and stained with EdU (green) and DAPI (blue) for brightfield (BF) and immunofluorescence analysis. Bar: 100μm. (B) Quantification of myoblast proliferation from the images of immunofluorescence staining for EdU. The percentiles of EdU positive cells in the view fields were counted as the readout of proliferation. Data was presented as mean ± SEM. (Student's *t-test*). N=3. (C) The mRNA levels of *Prr33* and proliferation marker genes (*Cdk1*, *Ccne1* and *etc*) in the myoblasts (cultured in growth medium) were assessed by qRT-PCR. Data was presented as mean ± SEM. (Student's *t-test*). Ctrl: N=4, *Prr33-*KD: N=6. (D) Representative images of C2C12 cells (differentiation day 3, D3) with immunofluorescence staining for MYH1E (MF20, green) and DAPI (blue). Bar: 100μm. (E) Myogenesis was assessed by quantifying myonuclei clustering in the cells. MF20 positive myotubes in each view field were counted by ImageJ (https://imagej.nih.gov/ij/), and grouped by their nuclei numbers (single nucleus, two to five nuclei, or greater than or equal to six nuclei). Myonuclei clustering was quantified as the percentiles of grouped myotubes. Data was presented as mean ± SEM. (Student's *t-test*). N=3. (F) The length of MF20 positive myotubes measured by ImageJ. Data was presented as mean ± SEM. (Student's *t-test*). N=3. (G) The expression of *Prr33* and differentiation marker genes at differentiation day 3 (D3) was assessed by qRT-PCR assay. Data was presented as mean ± SEM. (Student's *t-test*). Ctrl: N=6, *Prr33-*KD: N=4. (H) The expression of fast/slow twitch genes at differentiation day 3 (D3) was assessed by qRT-PCR assay (Fast-twitch: *Tnnt3*, *Myl9*, *Tpm2* and *Myl1*; Slow-twitch: *Tnnc1*, *Myl3*, and *Myh7b*). Data was presented as mean ± SEM. (Student's *t-test*). Ctrl: N=6, *Prr33-*KD: N=6.

**Supplemental figure S3. *Prr33* knock-down by CRISPR/Cas9 inhibits myoblast differentiation but has no influence on proliferation**

(A) Representative images of C2C12 myoblasts cultured in growth medium expressing Cas9/CRISPR-gRNA targeting *Prr33* gene (*Prr33*-gRNA) or Cas9/CRISPR-Ctrl-gRNA (Ctrl-gRNA) with immunofluorescence staining for EdU (green) and DAPI (blue). BF: bright filed. Bar: 100μm. (B) Quantification of myoblast proliferation from the images of immunofluorescence staining for EdU. The percentiles of EdU positive cells in the view fields were counted as the readout of proliferation. Data was presented as mean ± SEM. (Student's *t-test*). N=3. (C) Representative images of C2C12 myoblasts cultured in growth medium expressing Cas9/CRISPR-gRNA targeting *Prr33* gene (*Prr33*-gRNA) or Cas9/CRISPR-Ctrl-gRNA (Ctrl-gRNA) with immunofluorescence staining for Ki67 (green) and DAPI (blue). BF: bright filed. Bar: 100μm. (D) Quantification of myoblast proliferation from the images of immunofluorescence staining for Ki67. The percentiles of Ki67 positive cells in the view fields were counted as the readout of proliferation. Data was presented as mean ± SEM. (Student's *t-test*). N=3. (E) The length of MF20 positive myotube (at differentiation day 3, Fig.2C). Data was presented as mean ± SEM. (Student's *t-test*). N=3. (F) The length of MF20 positive myotube (at differentiation day 5, Fig.2E). Data was presented as mean ± SEM. (Student's *t-test*). N=3. (G) Myogenesis was assessed at differentiation day 3 (D3) by quantifying myonuclei clustering in the cells. MF20 positive myotubes in each view field were counted by ImageJ (https://imagej.nih.gov/ij/), and grouped by their nuclei numbers (single nucleus, two to five nuclei, or greater than or equal to six nuclei). Myonuclei clustering was quantified as the percentiles of grouped myotubes. Data was presented as mean ± SEM. (Student's *t-test*). N=3. (H) Myogenesis was assessed at differentiation day 5 (D5) by quantifying myonuclei clustering in the cells. MF20 positive myotubes in each view field were counted by ImageJ (https://imagej.nih.gov/ij/), and grouped by their nuclei numbers (single nucleus, two to five nuclei, or greater than or equal to six nuclei). Myonuclei clustering was quantified as the percentiles of grouped myotubes. Data was presented as mean ± SEM. (Student's *t-test*). N=3. (I) Ctrl-gRNA C2C12 and *Prr33*-gRNA C2C12 were cultured in growth medium (GM), in differentiation medium (DM 1day, DM 3days, DM 5days). Cell lysates were subjected to Western Blot analysis with indicated antibodies (anti-PRR33, anti-MF20 and anti-GAPDH). N=3. (J) Quantification of protein level of PRR33 and MF20 normalized to GAPDH (Fig.S3L). Data was presented as mean ± SEM. (Student's *t-test*). N=3. (K) The expression of fast/slow twitch genes at differentiation day 5 was assessed by qRT-PCR assay (Fast-twitch: *Tnnt3*, *Myl9*, *Tpm2* and *Myl1*; Slow-twitch: *Tnnc1*, *Myl3*, and *Myh7b*). Data was presented as mean ± SEM. (Student's *t-test*). Ctrl-gRNA: N=6, *Prr33-*gRNA: N=3.

**Supplemental figure S4. *Prr33* knock-down by siRNA inhibits myoblast differentiation but does not alert myotube atrophy**

(A) C2C12 were cultured in DM for 2 days and then transfected with siRNA targeting (si-*Prr33*). After *Prr33* was knocked-down, the cells were fixed and stained with MF20 antibody (green) and DAPI (blue) for immunofluorescence analysis. Representative images were shown. Bar:100μm. N=6. (B) Quantification of the percentage of MF20 negative myotubes showed in Fig.S4A. Data was presented as mean ± SEM. (Student's *t-test*). N=6. (C) The length of MF20 positive myotube (Fig.S4A) was measured by ImageJ. Data was presented as mean ± SEM. (Student's *t-test*). N=6. (D) C2C12 were cultured in DM for 2 days and then transfected with siRNA targeting (si-*Prr33*). qRT-PCR detected the expression of atrophy marker genes and differentiation marker genes. Data was presented as mean ± SEM. (Student's *t-test*). N=3.

**Supplemental figure S5. *Prr33* overexpression promotes myoblast differentiation**

(A) C2C12 myoblasts expressing *Prr33* (Ad-*Prr33*) or Lacz control (Ad-*Lacz*) were cultured in DM for 3 days. Cell lysates were subjected to Western Blot analysis with indicated antibodies. N=3. (B) Quantification of protein level of MYOD normalized by that of TUBULIN (Fig.S5A). Data was presented as mean ± SEM. (Student's *t-test*). N=3. (C) The length of MF20 positive myotubes (Fig. 2G) was measured through ImageJ. Data was presented as mean ± SEM. (Student's *t-test*). N=3. (D) Myogenesis was assessed by quantifying myonuclei clustering in the cells. MF20 positive myotubes in each view field were counted by ImageJ (https://imagej.nih.gov/ij/), and grouped by their nuclei numbers (single nucleus, two to five nuclei, or greater than or equal to six nuclei). Myonuclei clustering was quantified as the percentiles of grouped myotubes. Data was presented as mean ± SEM. (Student's *t-test*). N=3.

**Supplemental figure S6. Expression of genes encoding cell differentiation is altered in *Prr33^KO^* mouse muscles**

(A) The expression of differentiation markers in TA muscle were assayed by qRT-PCR. Data was presented as mean ± SEM. (Student's *t-test*). *Prr33^fl/fl^*: N=6, *Prr33^KO^*: N=6. (B) The expression of differentiation markers in TRI muscle were assayed by qRT-PCR. Data was presented as mean ± SEM. (Student's *t-test*). *Prr33^fl/fl^*: N=6, *Prr33^KO^*: N=6. (C) The expression of differentiation markers in QUAD muscle were assayed by qRT-PCR. Data was presented as mean ± SEM. (Student's *t-test*). *Prr33^fl/fl^*: N=6, *Prr33^KO^*: N=6. (D) The expression of differentiation markers in GAS muscle were assayed by qRT-PCR. Data was presented as mean ± SEM. (Student's *t-test*). *Prr33^fl/fl^*: N=6, *Prr33^KO^*: N=6. (E) The expression of differentiation markers in Soleus muscle were assayed by qRT-PCR. Data was presented as mean ± SEM. (Student's *t-test*). *Prr33^fl/fl^*: N=6, *Prr33^KO^*: N=6.

**Supplemental figure S7. *Prr33* deletion inhibits myoblast differentiation during regeneration**

(A) The expression of *Prr33* in skeletal muscle was assayed by qRT-PCR 3 days, 7 days, 14 days after BaCl_2_ injection. Ctrl: N=3, BaCl_2_-3d: N=3, BaCl_2_-7d: N=3, BaCl_2_-14d: N=5. (B) TA muscle of *Prr33^fl/fl^* and *Prr33^KO^* mouse injected with Saline or BaCl_2_ for 3 days. Tissue lysates were subjected to Western Blot analysis with indicated antibodies. (C) Quantification of eMHC protein level in the muscle samples collected 3 days after injection BaCl_2_. *Prr33^fl/fl^*-BaCl_2_-3d: N=6; *Prr33^KO^* -BaCl_2_-3d: N=5. (D) Cross-section areas (CSA) of eMHC positive TA myofibers from *Prr33^fl/fl^* or *Prr33^KO^* mouse were analyzed at 7 days after BaCl_2_ injection using ImageJ. Data was presented as mean ± SEM. (Student's *t-test*). N=3. (E) Cross-section areas (CSA) of eMHC positive TA myofibers from *Prr33^fl/fl^* or *Prr33^KO^* mouse were analyzed at 14 days after BaCl_2_ injection using ImageJ. Data was presented as mean ± SEM. (Student's *t-test*). N=3. (F) H&E staining of cross sections of *Prr33^fl/fl^* and *Prr33^KO^* TA muscle after BaCl_2_ injection 14 days. N=3. Bar: 100μm. (G) Gross morphology of *Prr33^fl/fl^* and *Prr33^KO^* TA muscles at day 14 post BaCl_2_ injection. N=3. Bar: 25mm.

**Supplemental figure S8.** **Expression of genes encoding cell differentiation, cytoskeleton and mitochondrial factors are altered in *Prr33^KO^* mouse muscle**

(A) qRT-PCR assay for genes encoding muscle differentiation, cytoskeleton and mitochondrial factors in *Prr33^fl/fl^* and *Prr33^KO^* mouse TA muscle. Data was presented as mean ± SEM. (Student's *t-test*). *Prr33^fl/fl^*: N=4, *Prr33^KO^*: N=3.

**Supplemental figure S9. *Prr33* ablation alters the ultrastructure of mitochondria**

(A) Z-line length was measured from TEM images with ImageJ and quantified. N=3. Data was presented as mean ± SEM. (Student's *t-test*). (B) The number of cristae was counted and normalized to the number of mitochondria. N=3 (quantified mitochondria numbers: *Prr33^fl/fl^* (100); *Prr33^KO^* (100)). Data was presented as mean ± SEM. (Student's *t-test*). (C) Cristae length was measured from TEM images with ImageJ and quantified. N=3 (quantified cristae numbers: *Prr33^fl/fl^* (207); *Prr33^KO^* (182)). Data was presented as mean ± SEM. (Student's *t-test*). (D) Mitochondrial area was measured from TEM images with ImageJ and quantified. N=3 (quantified mitochondria numbers: *Prr33^fl/fl^* (206); *Prr33^KO^* (203)). Data was presented as mean ± SEM. (Student's *t-test*).

**Supplemental figure S10. *Des* knock-down inhibits myoblast differentiation and impairs mitochondrial function**

(A) DESMIN was knocked down in C2C12 by siRNA (si-*Des*) and control (si-NC). Cells were fixed at differentiation day 3 and stained with MF20 antibody (green) and DAPI (blue) for immunofluorescence analysis. Bar:100μm. (B) Myogenesis was assessed by quantifying myonuclei clustering in the cells. MF20 positive myotubes in each view field were counted by ImageJ (https://imagej.nih.gov/ij/), and grouped by their nuclei numbers (single nucleus, two to five nuclei, or greater than or equal to six nuclei). Myonuclei clustering was quantified as the percentiles of grouped myotubes. Data was presented as mean ± SEM. (Student's *t-test*). N=3. (C) The length of MF20 positive myotube was measured with ImageJ. Data was presented as mean ± SEM. (Student's *t-test*). N=3. (D) The expression of *Des* and differentiation markers at differentiation day 3 was assessed by qRT-PCR. Data was presented as mean ± SEM. (Student's *t-test*). N=3. (E) DESMIN was knocked down in C2C12 by siRNA (si-*Des*). Cell lysates were subjected to Western Blot analysis with indicated antibodies. N=3. (F) Quantification of protein level of DESMIN and PRR33 normalized to GAPDH (Fig.S10E). Data was presented as mean ± SEM. (Student's *t-test*). N=3. (G) Mitochondrial respiration assay for C2C12 cells transfected with si-RNA (si-NC or si-*Des*). Mitochondrial function was measured at differentiation day 3. (H) Basal OCR in myotubes. Data was presented as mean ± SEM. (Student's *t-test*). (I) Maximal OCR in myotubes. Data was presented as mean ± SEM. (Student's *t-test*).

**Supplemental figure S11. *Prr33* deletion affects DESMIN accumulation and mitochondrial morphology after BaCl_2_ injection 14 days**

(A) Immunostaining of *Prr33^fl/fl^* and *Prr33^KO^* TA muscle at 14 days after BaCl_2_ injection with anti- DESMIN antibody (green), DAPI (blue) and WGA (white). White arrows: accumulated DESMIN. N=3. Bar: 40μm. (B) The percentiles of myofibers with abnormal DESMIN accumulation in TA muscles at 14 days after BaCl_2_ injection. Data was presented as mean ± SEM. (Student's *t-test*). N=3.

**Supplemental figure S12.** ***Prr33* ablation alters DESMIN-mitochondria contacts and patterning during differentiation**

(A) Ctrl-gRNA and *Prr33*-gRNA-treated C2C12 myoblast (GM) stained with anti-TOMM20 antibody (green) and DAPI. Representative images were shown. Bar: 30μm. (B) Immunostaining of *Prr33^fl/fl^* and *Prr33^KO^* TA muscle fibers with anti- DESMIN antibody (green), anti-PRR33 (red) and DAPI (blue). Asterisk (*): accumulated DESMIN. N=3. Bar: 40μm. (C) Immunostaining of *Prr33^fl/fl^* and *Prr33^KO^* TA muscle fibers with anti-TOMM20 antibody (green), anti-PRR33 (red) and DAPI (blue). White arrows: disorganized mitochondria. N=3. Bar: 40μm. (D) Immunostaining of *Prr33^fl/fl^* and *Prr33^KO^* TA muscle fibers with anti-DESMIN antibody (green), anti-TOMM20 (red) and DAPI (blue). Asterisk (*): accumulated DESMIN; White arrows: disorganized mitochondria. N=3. Bar: 40μm. (E) Percentiles of myofibers with abnormal accumulated DESMIN in *Prr33^fl/fl^* and *Prr33^KO^* TA muscle fibers. N=3 mice for each group. (quantified myofiber numbers: *Prr33^fl/fl^* (90); *Prr33^KO^* (88). (F) Percentiles of myofibers with disorganized mitochondria in *Prr33^fl/fl^* and *Prr33^KO^* TA muscle fibers. (quantified myofiber numbers: *Prr33^fl/fl^* (82); *Prr33^KO^* (89). (G) Gel analysis of biotinylated DESMIN “neighbor Proteins”. Ctrl-gRNA and *Prr33*-gRNA-treated C2C12 myoblasts were infected by Ad-Turbo-*Des*-flag (lanes 2-3,5-6) and cultured in DM for 2 days. Then incubate the cells with medium containing biotin to label DESMIN “neighbor Protein” for 0 minute (lanes 2 and 5), for 25 minutes (lanes 1, 3, 4 and 6). M= Protein Marker. (H) Western Blot analysis of the mitochondrial protein TUFM labeled by Turbo-ID.

**Supplemental Table legends**

**Supplemental table 1. gRNAs used to generate knockout C2C12**

**Supplemental table 2. Primers for genotyping mouse and CRISPR/Cas9 knockout cells**

**Supplemental table 3. Sequences for shRNA and siRNAs**

**Supplemental table 4. Primers for real-time PCR used in this study**

**Supplemental table 5. Antibodies used in this study**
